# A Multifunctional Mutagenesis System for Analysis of Gene Function in Zebrafish

**DOI:** 10.1534/g3.114.015842

**Published:** 2015-04-02

**Authors:** Helen Ngoc Bao Quach, Shijie Tao, Pavle Vrljicak, Adita Joshi, Hua Ruan, Rashmi Sukumaran, Gaurav K. Varshney, Matthew C. LaFave, The Ds Screen Team, Shawn M. Burgess, Christoph Winkler, Alexander Emelyanov, Sergey Parinov, Karuna Sampath

**Affiliations:** * Temasek Life Sciences Laboratory, National University of Singapore, Singapore 117604; † Department of Biological Sciences, National University of Singapore, Singapore 117543; ‡ Division of Biomedical Cell Biology, Warwick Medical School, Coventry, United Kingdom CV4 7AJ; § National Human Genome Research Institute, National Institutes of Health, Bethesda, MD 20892-8004; ** Centre for Bioimaging Sciences, National University of Singapore, Singapore 117543

**Keywords:** functional genomics, insertional mutagenesis, *Ac/Ds* transposon, gene expression

## Abstract

Since the sequencing of the human reference genome, many human disease-related genes have been discovered. However, understanding the functions of all the genes in the genome remains a challenge. The biological activities of these genes are usually investigated in model organisms such as mice and zebrafish. Large-scale mutagenesis screens to generate disruptive mutations are useful for identifying and understanding the activities of genes. Here, we report a multifunctional mutagenesis system in zebrafish using the maize *Ds* transposon. Integration of the *Ds* transposable element containing an mCherry reporter for protein trap events and an EGFP reporter for enhancer trap events produced a collection of transgenic lines marking distinct cell and tissue types, and mutagenized genes in the zebrafish genome by trapping and prematurely terminating endogenous protein coding sequences. We obtained 642 zebrafish lines with dynamic reporter gene expression. The characterized fish lines with specific expression patterns will be made available through the European Zebrafish Resource Center (EZRC), and a database of reporter expression is available online (http://fishtrap.warwick.ac.uk/). Our approach complements other efforts using zebrafish to facilitate functional genomic studies in this model of human development and disease.

Although at least 20,000 protein-coding genes have been identified in the human genome, only a small number of genes have been well-studied, and the normal function or disease significance of many genes remains obscure (Edwards *et al.* 2011). Due to low spontaneous mutation frequency and other ethical considerations pertaining to research in humans, large-scale mutagenesis in model organisms is the most efficient way to discover novel genes and generate tools to dissect genetic pathways in human diseases and development. It is important to assemble genetic resources from multiple organisms to facilitate comprehensive understanding of biological activities of genes, and the well-annotated genome sequences of many organisms have provided a strong foundation for genome-wide genetic screens (White *et al.* 2013). Recently, the zebrafish genome was completely sequenced and its relationship to the human genome has been revealed, indicating the value of this model organism for functional analysis of vertebrate genes and, in particular, human disease genes. Several recent efforts have aimed to systematically mutate all protein-coding genes in zebrafish (Howe *et al.* 2013; Kettleborough *et al.* 2013; Varshney *et al.* 2013; Miller *et al.* 2013). In large-scale mutagenesis screens using the chemical mutagen, N-ethyl-N-nitrosourea (ENU), a number of mutants were identified for many known zebrafish protein-coding genes, aided by high-throughput sequencing methods and a well-annotated zebrafish reference genome (Kettleborough *et al.* 2013; Miller *et al.* 2013; Driever *et al.* 1996; Haffter *et al.* 1996). A Moloney murine leukemia virus (MMLV)-based insertion mutagenesis strategy has also isolated thousands of zebrafish mutations (Varshney *et al.* 2013). These mutants are valuable tools for the study of their human orthologs.

Protein trapping offers an alternative, powerful approach to abolish gene function by random insertion of DNA. A protein trap construct typically contains a splice acceptor site immediately upstream of a promoter-less reporter gene to create reporter-tagged fusion proteins. This approach simultaneously mutates the trapped gene and provides information about its *in vivo* expression (Gossler *et al.* 1989; Kawakami *et al.* 2004b; Skarnes *et al.* 1992; Skarnes *et al.* 2004; Trinh le *et al.* 2011). However, enhancer trap (ET) vectors contain a weak basal promoter that requires the cassette to insert in the vicinity of *cis*-acting enhancer elements to express the reporter gene under the control of endogenous sequences. The ET reporter is expressed in a spatio-temporal fashion under the control of endogenous enhancers, providing dynamic gene expression information. However, this reporter system does not efficiently create mutants (Allen *et al.* 1988; Kothary *et al.* 1988; O’Kane and Gehring 1987; Weber *et al.* 1984).

Various gene trap and enhancer trap vectors have been applied in animal model organisms, such as *Drosophila*, mice, zebrafish, and the Japanese rice fish medaka, successfully capturing the functional proteome and enabling visualization of fluorescent reporter expression regulated by endogenous elements (Hummel and Klambt 2008; Lukacsovich and Yamamoto 2001; Morin 2003; Gossler *et al.* 1989; Stanford *et al.* 2001; Wurst *et al.* 1995; Asakawa and Kawakami 2009; Froschauer *et al.* 2012; Kawakami *et al.* 2004b; Trinh le *et al.* 2011; Clark *et al.* 2011; Grabher *et al.* 2003). Trapping vectors can be efficiently introduced into genomes by electroporation, microinjection, or retroviral infection, depending on the vector design and model system. Electroporation can lead to tandem insertions into the same locus, and vector DNA is often digested by exonucleases, making the cloning of insertion sites problematic (Stanford *et al.* 2001). Retroviral vectors have a tendency to insert into the 5′ region of genes, and their packaging size is limited (Stanford *et al.* 2001). DNA transposon-based protein trap and enhancer trap systems overcome some of these disadvantages and provide additional tools for efficient genome engineering. The first widely used DNA transposon was the *P* element in *Drosophila* (Rubin and Spradling 1982; Spradling and Rubin 1982). Then, an active hAT family DNA transposon *Tol2* was identified and cloned from medaka (Koga *et al.* 1996; Parinov *et al.* 2004) and subsequently used for gene transfer in many vertebrate genomes, including zebrafish, frog, chicken, mouse embryonic stem cells, and human cells (Kawakami 2005, 2007; Kawakami *et al.* 2004a,b; Parinov *et al.* 2004; Hamlet *et al.* 2006; Kawakami and Noda 2004; Sato *et al.* 2007; Tanabe *et al.* 2006; Wu *et al.* 2006). The *Tc1/mariner* family transposon, *Sleeping Beauty (SB)*, which was reconstructed from fish, and the *piggyBac (PB)* transposon, reconstructed from moths, are also commonly used transposons that have been used for insertional mutagenesis and genetic manipulation in fish, frogs, and mammalian cells (Grabher *et al.* 2003; Ivics *et al.* 1997; Kitada *et al.* 2007; Mates *et al.* 2009; Munoz-Lopez and Garcia-Perez 2010; Sinzelle *et al.* 2006; Wilson *et al.* 2007; Yusa *et al.* 2009). Recently, the first transposon to be discovered, the Activator (Ac)/Dissociation (Ds) system from maize, has also been successfully adopted for germ-line transgenesis in vertebrates and has been demonstrated to be an effective gene transfer vehicle (Boon Ng and Gong 2011; Emelyanov *et al.* 2006; Froschauer *et al.* 2012; Trinh le *et al.* 2011; McClintock 1948). To utilize the strengths of gene and enhancer trapping, we developed a multifunctional DsDELGT4 system to simultaneously introduce both protein trap and enhancer trap cassettes into the zebrafish genome using the maize *Ac/Ds* transposon system. Compared with single protein trap or enhancer trap systems, our dual trap system not only provides expression information but also creates protein-disrupting mutants in the same screen.

*Ac/Ds* belongs to the large hAT family and acts by the "cut-and-paste" mechanism (McClintock 1948, 1951). The *Ac* or Activator is an autonomous element carrying a transposase gene encoded between the *cis*-required terminal sequences. The *Ds* or Dissociation element does not harbor sequences encoding the transposase, but contains the *cis*-required terminal sequences, which can be *trans*-activated only in the presence of the *Ac* element or Ac transposase (McClintock 1951). The *Ac/Ds* system has been used for mutagenesis in many plant species and has also been demonstrated to function in vertebrate species and cultured human cells (Boon Ng and Gong 2011; Chin *et al.* 1999; Emelyanov *et al.* 2006; Froschauer *et al.* 2012; Kuromori *et al.* 2004). Several studies have reported that the *Ac/Ds* system can achieve high germ-line transmission rates in both medaka and zebrafish embryos by microinjection at the one-cell stage (Boon Ng and Gong 2011; Emelyanov *et al.* 2006).

We screened 2790 F0 founders and successfully obtained 642 zebrafish lines with fluorescent reporter expression. Each line contains, on average, 4–6 *Ds* transposon insertions. We used the thermal asymmetric interlaced polymerase chain reaction (TAIL-PCR) method to identify genomic sequences flanking the *Ds* insertions. Here, we report the identification of 277 integration sites that can be unambiguously mapped to the zebrafish genome (Zv9). In addition, we provide reporter expression information for the transposon lines and some representative mutant phenotypes. These lines will be made available to the research community via the EZRC. Our multifunctional *Ds* transposon collection can be a useful resource for analyzing gene expression and function in this vertebrate model of development and disease.

## Materials and Methods

### Transposon vectors

The pDsDELGT4 donor construct was derived from the pDs vector containing the 5′*Ds* and 3′*Ds* ends (Emelyanov *et al.* 2006). Downstream of the 5′*Ds* element, zebrafish B-cell CLL/lymphoma 2 (*bcl2*) splice acceptor site sequence (663 bp) was cloned by PCR, based on the zebrafish *bcl2* gene intron 1/exon 2 boundary (ENSDARG00000089109), and inserted before *mCherry* (lacking the first methionine) sequence, which was followed by bovine growth hormone polyadenylation (BGH-PolyA) signal sequence. In the opposite orientation, downstream of the 3′*Ds* repeat sequences, a short *glial fibrillary acidic protein* (GFAP) promoter (consisting of 360 bp of upstream sequence and the entire 16 bp 5′UTR) was sub-cloned into the pDs vector, preceding a lox2272 site and EGFP sequences, and followed by an SV40 polyA signal. The vector design excluded the GFAP enhancer driving glial expression, and no specific CNS activity was observed in transient assays. A mini-*tol2* cassette was placed between the BGH polyA and SV40 polyA signal. Ac transposase mRNA was synthesized *in vitro* using the mMESSAGE mMACHINE SP6 Kit (Ambion, AM1340M) from the NLS^K5E^-TPase construct (Emelyanov *et al.* 2006).

### Microinjection

Aliquots of donor pDsDELGT4 plasmid DNA (100 pg) were co-injected with 100 pg of transposase mRNA into one-cell stage AB zebrafish embryos. Injected embryos showing reporter expression at 24 hr after fertilization were raised to adulthood as the F0 generation. For remobilization of *Ds* insertions, 100 pg of transposase mRNA was injected into embryos from intercrosses of homozygous *Tg(DsDELGT4)ws0310* or *Tg(DsDELGT4)ws01961* parent pairs. For *Tg (DsDELGT4)ws0310*, to detect excision events at the integration locus on chromosome 21, primers 5′-TCCAAGTGATCAACTAGAAGTC-3′ and 5′-GAGACTTGCACTGATATACTGACTG-3′ were used to amplify sequences surrounding the original integration site. PCR products were cloned into the pGEM-T easy vector (Promega, A1360) for sequencing.

### Screening of transgenic fish

F0 founders were raised to adulthood and crossed with wild-type zebrafish for testing the germ-line transmission rates by observing EGFP and mCherry expression in F1 embryos from 0–7 d after fertilization. Reporter negative embryos were collected for *Ds*-specific PCR (5′-TCAAGCGGCCGCCTGTGTTTCAGACA-3′ and 5′-ACCGTTTCACCGGGATCCCGTTTTTAA-3′) to test for the presence of *Ds* integrations without fluorescent reporter expression. Reporter expression-positive embryos were raised to adulthood and named according to ZFIN nomenclature guidelines (Mullins 1995) after the founder number, such that the laboratory allele designation, ws, is followed by a four-digit unique founder number, for example, *Tg(DsDELGT4)ws1234*. Founders were numbered sequentially in the order that they were identified. For lines with multiple insertions, each insertion was given another identifier digit after the four-digit founder number, for example, *Tg(DsDELGT4)ws12341* or *Tg(DsDELGT4)ws12342*, such that each integration has a unique identity. New lines derived from a particular line by remobilization or by excision were given an additional letter suffix. For example, lines derived from *Tg(DsDELGT4)wsXXXX* or *Tg(DsDELGT4)wsXXXX1* were named *Tg(DsDELGT4)wsXXXXa* or *Tg(DsDELGT4)wsXXXX1a*.

### Microscopy

Embryos were anesthetized in 0.168 mg/ml Tricaine methanesulfonate (Sigma, A-5040) and mounted in 0.8–1% low-melting agarose gel (Bio-Rad, 161-3111) in egg water before observation. GFP and mCherry reporter expression was observed and the various patterns were recorded under either a Nikon Eclipse 80i upright microscope or a Zeiss Axiovert inverted fluorescence imaging system, equipped with a Coolsnap monochrome camera (Photometrics, EZ). Higher magnification images were documented using a Leica SP5 TCF inverted confocal microscope.

### Genomic DNA preparation

DNA from caudal fins excised from adult fish or from whole single embryos was isolated in 400 µl or 30 µl of DNA lysis buffer (0.1 M Tris, pH 8, 0.1 M NaCl, 0.05 M EDTA, 0.5% SDS) containing 100 µg/ml proteinase K (Promega, V3021) for 10 hr at 55° and then incubated for 10 min at 65° to heat-inactivate the proteinase K. DNA was purified using phenol-chloroform and precipitated using isopropanol. DNA pellets were washed with 70% ethanol and resuspended in water.

### Southern blot analysis

Caudal fins were lysed as described above and genomic DNA was extracted using phenol-chloroform, precipitated with isopropanol, and re-suspended in 30 µl of Tris-EDTA (TE) buffer. Ten mg of genomic DNA was digested overnight with *Sac* II and *Nde* I, separated on a 0.8% agarose gel (Bio-Rad, 161-3102), followed by transfer onto a Amersham Hybond N+ nylon membrane (GE Healthcare) and UV cross-linking (Stratagene, UV Stratalinker 1800). Antisense *mCherry* DNA probe was labeled with digoxigenin (DIG) by using the PCR DIG probe synthesis kit (Roche, 11636090910). The EasyHyb DIG wash and block buffer set was used for hybridization (Roche, 11585762001), and anti-DIG-AP Fab fragments (Roche, 1093274) and CDP-Star chemiluminescent substrate (Roche, 11685627001) were used to detect the hybridized probes.

### Cryopreservation and *in vitro* fertilization

Sperm from individual killed males was released in 100 µl of FBS (Sigma, F0926), containing 15% N, N-dimethylacetamide (Sigma, D5511), and was mixed briefly by pipetting. Four aliquots of 25 µl each were pipetted directly into 2-ml cryovials (Simport, T311-2) and transferred immediately into a pre-chilled 50-ml Falcon tube on dry ice. After 20 min, the samples were moved into liquid nitrogen for at least 1 hr and stored long-term in liquid nitrogen. For *in vitro* fertilization, sperm samples were thawed by adding 500 µl FBS at 37° into the frozen cryovials. The sperm suspension was mixed immediately with freshly squeezed eggs in a Petri dish and activated by addition of 0.1 ml of I-buffer (116 mM NaCl, 23 mM KCl, 6 mM CaCl_2_, 2 mM MgSO_4_, 29 mM NaHCO_3_, and 0.5% fructose). After 30 sec, another 0.25 ml of 0.5% fructose egg water was added into the dish. Fertilization rates were checked at 3 hr after fertilization at 28.5°.

### Mapping of *Ds* integration sites

#### TAIL-PCR and analysis of flanking sequence:

TAIL-PCR was performed as previously described (Liu and Whittier 1995; Parinov *et al.* 2004; Emelyanov *et al.* 2006) using the following set of primers:

*Ds*5′-1, 5′-TAGAAAATACGGTAACGAACGGGATCATCC-3′;
*Ds*5′-2, 5′-CCGTTTACCGTTTTGTATATCCCG-3′;
*Ds*5′-3, 5′-TCGTTTTTTACCTCGGGTTCGAAATCG-3′;
*Ds*3′-1, 5′-ATGTTAGCCAAGAGCCCAAGACTTATCAC-3′;
*Ds*3′-2, 5′-CAAAAATACCGGTTCCCGTCCGATTT-3′;
*Ds*3′-3, 5′-CGATTACCGTATTTATCCCGTTCG-3′;
AD5, 5′-WCAGNTGWTNGTNCTG-3′;
AD11, 5′-NCASGAWAGNCSWCAA-3′;
AD6, 5′-STTGNTASTNCTNTGC-3′ and AD3-2, 5′-NGTASASWGTNAWCAA-3′.

Products of secondary and tertiary PCRs were separated on 1.8% agarose gels. Individual fragments from “band shift” pairs were sliced from the gel, purified using the QIAquick gel extraction kit (QIAGEN), and sequenced with *Ds*5′-3 and *Ds*3′-3 primers. Flanking sequences obtained from TAIL-PCR were analyzed against the zebrafish reference genome (Zv9) and Ensembl gene databases. BLAT was used to map the sequences to the reference genome. Flanking sequences were considered unambiguously mapped if the sequence obtained by TAIL-PCR matched a given location of the genome assembly with 85% identity or more.

To determine insertion sites for lines that could not be resolved by TAIL-PCR, high-throughput sequencing was performed on the Illumina MiSeq as described previously (Varshney *et al.* 2013) with the following modifications. The first round of PCR was performed using a 3′ Ds ITR primer and a linker primer (5′-TATGAAAATGAAAACGGTAGAGGTATTTTACCGACCG-3′ and 5′-GTAATACGACTCACTATAGGGCACGCGTG-3′, respectively) and the second round of PCR was performed using nested 3′ Ds ITR and linker primers (5′-TTTACCGACCGTTACCGACCGTTTTCATC-3′ and 5′-GCGTGGTCGACTGCGCAT-3′, respectively). Insertion sites were identified using a version of the GeIST program that had been modified to detect Ds integrations (Lafave *et al.* 2014). Sequence logos were generated using weblogo v3 (Crooks *et al.* 2004

### Rapid amplification of cDNA ends (RACE)

For 5′ RACE to identify trapped genes upstream of the mCherry reporter, we used the First Choice RLM Race kit from Ambion (Ambion, AM1700). Typically, 10 μg of total RNA was extracted using Trizol (Invitrogen) and dephosphorylated with calf intestine alkaline phosphatase (CIP). The cap structure of full-length mRNA is not affected by CIP, but it is removed with tobacco acid pyrophosphatase (TAP) before ligation to a 45-base RNA Adapter using T4 RNA ligase. During the ligation reaction, the majority of full-length de-capped mRNA acquires the adapter sequence as its 5′ end. First-strand cDNA was synthesized in a random decamer primed reverse-transcription reaction, and nested PCR with primers to *mCherry* and the 5′ RACE adapter were used to amplify the 5′ end of trapped genes. For the first round of amplification, we used the *mCherry* external primer (5′-CTTGTAGATGAACTCGCCGTCCTG-3′) and 5′ RACE outer primer (5′-GCTGATGGCGATGAATGAACACTG-3′). For the second round of amplification, we used the *mCherry* inner primer (5′-AGCTTCAAGTAGTCGGGGATGTCG-3′) and 5′ RACE inner primer (5′-CGCGGATCCGAACACTGCGTTTGCTGGCTTTGATG-3′). PCR was performed as instructed by the manufacturer, and PCR products were gel-purified and cloned into the pGEM-T easy vector for sequencing.

### Validation of integration sites

Once an integration site was identified, forward and reverse genomic primers were designed to sequences either upstream or downstream of the integration locus. The 5′ *Ds* or 3′ *Ds* primers were used in combination with genomic primers according to the orientation of the *Ds* inserts. For verifying *Ds* insertions in the sense orientation, we used 5′ *Ds* reverse primer (5′-ACCTTGTATGGCTCGAGGGATC-3′) and 3′ *Ds* forward primer (5′-AGTTACTCCGGAGTTGCTCTGC-3′); for verifying *Ds* insertions in antisense direction, we used 5′ *Ds* forward primer (5′-AGTAGCGTGTACTGGCATTAGATTG-3′) and 3′ *Ds* reverse primer (5′-AGCTTGATATCGAATTCCTGCAGC-3′).

### Morpholino and RNA injections

All morpholinos were obtained from GeneTools. To reduce side effects and lethality, *dhx37* ATG morpholinos and *dhx37* intron 4/exon 5 splice morpholinos were co-injected with *p53* morpholino at a concentration of 1+1.5 ng per embryo. The morpholino sequences are:

*dhx37* ATG morpholino: 5′-TGTGTTTCTTTCTCAATCTGCCCAT-3′
*dhx37* intron4/exon5 morpholino: 5′-TCTGCAAGAACACAGCAAAAAACAC-3′.

The cDNA encoding full-length *dhx37* gene was amplified by PCR and cloned into the pCS2+ vector. For injection, constructs were digested with *Not I* and the capped mRNA was synthesized with the mMessage mMachine SP6 kit (Ambion, AM1340) according to the manufacturer’s instructions. For rescue experiments, 200 pg of *dhx37* mRNA was injected into *dhx37^ws0977Tg/+^* intercross embryos.

### Detection of cell death, lipids, and RNA expression

Acridine Orange was used to detect cell death. Live embryos were incubated for 30 min at 28° in egg water containing 5 μg/ml Acridine Orange (Sigma). After extensive washes in egg water, embryos were anesthetized in tricaine (Sigma) and imaged using a fluorescence microscope.

Oil red O was used to visualize lipids. Embryos were fixed in 4% paraformaldehyde/PBS. Oil Red O was applied overnight in 100% propylene glycol. After staining, embryos were washed in 85% propylene glycol and PBS before imaging.

Whole-mount *in situ* hybridization was performed as described (Tian *et al.* 2003) with a *flk1* probe (Stainier *et al.* 1995) on embryos fixed in 4% paraformaldehyde/PBS.

## Results

### Visualization and identification of enhancer trap and protein trap fluorescent reporters

**Figure 1:**
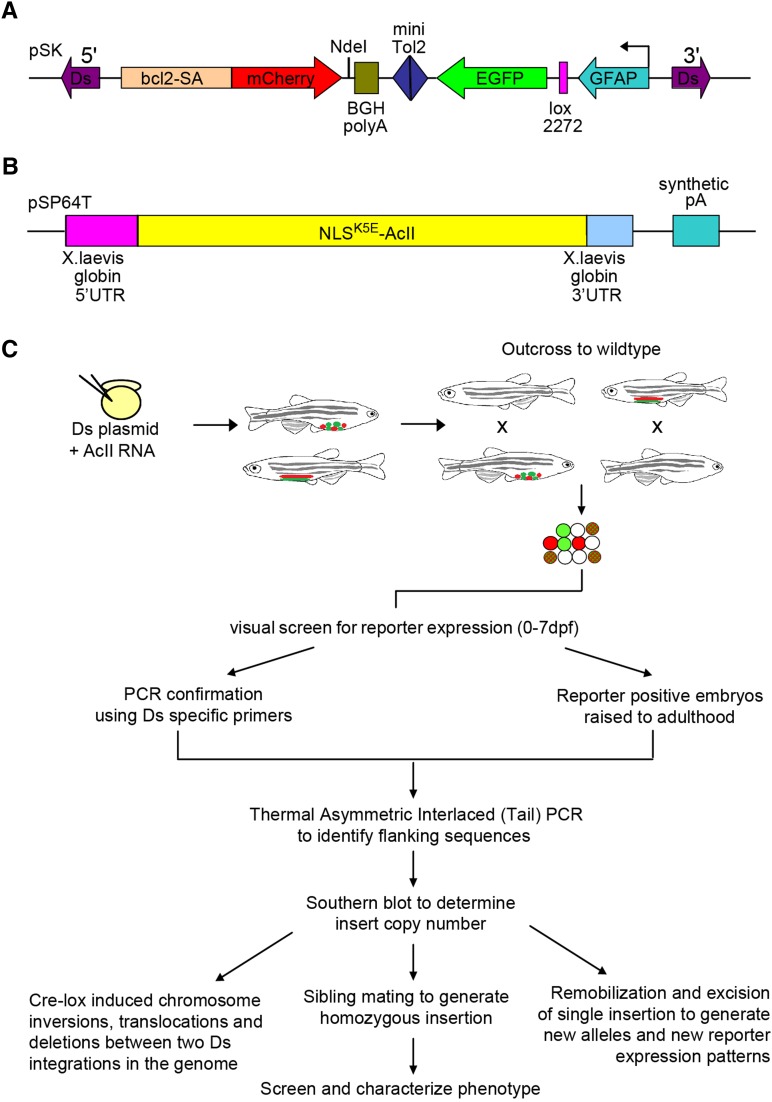
Schematic representation of the multi-functional *Ac/Ds* transposon system and insertion screen. (A) The pDsDELGT4 vector consists of a protein trap unit and an enhancer trap unit. The protein trap unit is close to the *Ds* 5′ terminal repeat sequences. The *mCherry* coding sequence without the first methionine (red) is flanked by the zebrafish *B-cell leukemia/lymphoma 2 (bcl2)* splice acceptor sequence and the bovine growth hormone (BGH) polyadenylation signal. In the reverse orientation, close to the *Ds* 3′ terminal repeat sequences, the enhancer trap reporter GFP (green) is downstream of a short *glial fibrillary acidic protein* (GFAP) promoter and a lox2272 site. A mini *Tol2* sequence is present between the two trap units (blue). (B) Schematic representation of the construct for synthesizing Ac transposase mRNA, with 5′UTR and 3′UTR sequences from the *Xenopus globin* gene. (C) Overview of the *DsDELGT4* mutagenesis screen. pDsDELGT4 plasmid was co-injected with capped Ac mRNA into one-cell stage embryos (F0). Founders with transient reporter expression were raised to adulthood and mated with wild-type (AB) fish. F1 embryos were visually screened for reporter expression from fertilization until 7 d after fertilization. *Ds* integrations were verified by PCR using *Ds* specific primers. Reporter positive F1 embryos were raised to adulthood. TAIL-PCR and Southern hybridization were performed with genomic DNA isolated from the tail-fin of F1 fish and subsequent generations to map the integrations and determine the number of *Ds* insertions. Phenotype analysis of homozygous mutants generated by mating siblings with the same integration was performed. Cre-mediated recombination between two *Ds* integrations was performed to generate precise segmental deletions.

In this multi-functional screen, we developed a protein trap and enhancer trap mutagenesis cassette flanked by 5′ and 3′ terminal sequences of the *Ds* element, allowing integration in the presence of the *Ac* transposase. The pDsDELGT4 vector has several features that allow it to efficiently report gene expression and/or disrupt gene function. The vector contains two key domains that are used to capture genomic sequences that encode enhancers and protein coding genes. The enhancer and protein trap cassettes are in opposite orientation, with an intervening mini *Tol2* element, which can act as a launching pad for Tol2 transposase-mediated remobilizations in the vicinity of the targeted *DsDELGT4* insertion sites, for further insertional mutagenesis (Figure 1A).

In the protein trap cassette, a zebrafish B-cell CLL/lymphoma 2 (*bcl2*) splice acceptor sequence (at the intron 1/exon 2 junction of transcript ENSDART00000128843) was placed immediately upstream of the *mCherry* reporter gene, which lacks a translation start site but maintains the stop codon sequence, followed by the bovine growth hormone (BGH) poly-adenylation signal. When *DsDELGT4* integrates into an intron in the same orientation as the protein coding sequence [open reading frame (ORF)], the *bcl2* splice acceptor sequence creates a fusion transcript between endogenous upstream exons and *mCherry* sequences, either by competing with normal splicing or by, perhaps, preventing it. Protein translation terminates after *mCherry* sequences and expression of the truncated mCherry fusion protein is only detected when *mCherry* is spliced in-frame with endogenous coding sequences. In the enhancer trap cassette, the GFP reporter is controlled by a short *glial fibrillary acidic protein* (GFAP) promoter that is expressed when the *DsDELGT4* insertion is near enhancer sequences. In addition, there is a lox2272 site between the GFAP and GFP sequences. When there are two insertions in the genome, injection of RNA encoding Cre recombinase can induce defined and precise chromosomal deletions, inversions, or translocations, depending on the location and orientation of the lox2272 sites (Figure 1A).

To generate transgenic lines, we co-injected the pDsDELGT4 vector and capped mRNA encoding *Ac* transposase (Figure 1B) into one-cell stage zebrafish embryos. At the doses used, 50% of the injected embryos survived until 24 hr after fertilization, when we screened embryos for reporter gene expression. Typically, 100% of the injected embryos that survived showed transient GFP expression in various cell types, whereas transient mCherry expression was rarely observed. Embryos that exhibited transient reporter expression were raised to adulthood as F0 founders.

Each F0 founder fish was mated with wild-type fish to produce F1 embryos. We performed a visual screen on F1 embryos at various early developmental stages starting from fertilization until 7 d after fertilization. Reporter-positive progeny from each founder were raised as a separate line named after the founder number (Figure 1C). In total, we screened 2790 F0 individuals and identified 26 lines expressing only mCherry, 278 lines expressing only GFP, and 338 lines expressing both mCherry and GFP. The germ-line transmission rate based on reporter expression is 23% (Table 1). However, not every *Ds* insertion will cause trap events or show reporter expression within the first 7 d after fertilization, and many protein traps are also likely to be out of the reading frame and may not produce mCherry fusion proteins. Therefore, the actual germ-line transmission rate for our *DsDELGT4* transposon is likely to be higher than 23%. In fact, we collected genomic DNA from F1 embryos lacking reporter expression and examined the presence of *Ds* insertions by PCR (Figure 1C). An additional 176 lines were found to be PCR-positive for *Ds* insertions, even though they did not express either of the reporter genes at the stages examined. Therefore, the actual germ-line transmission frequency for *DsDELGT4* is ∼30%.

**Table 1:**
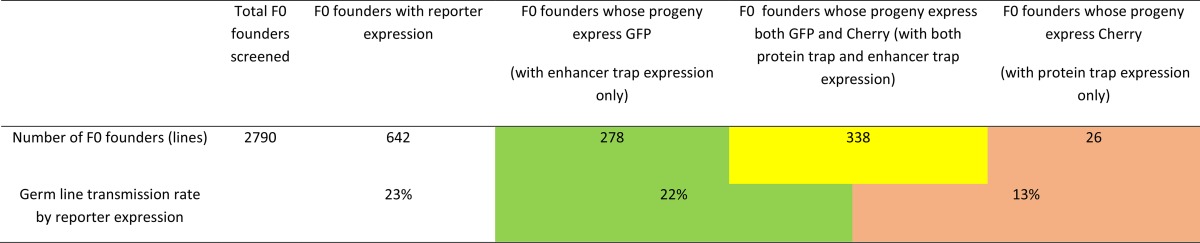
Screen summary

We often observed different reporter expression patterns in embryos derived from the same F0 founder, suggesting the Ac transposase introduces multiple *Ds* insertions in the germ cells of founders. Southern analysis performed using a *mCherry* probe on genomic DNA from 42 representative F1 fish or their progeny showed that the founders harbor multiple *Ds* insertions, ranging from one to eight copies (Supporting Information, Figure S1). We used TAIL-PCR to identify the sequences flanking the integration sites and mapped 277 sequences to the zebrafish reference genome (Zv9 assembly). In cases where we failed to identify any insertions or where the insert numbers from TAIL-PCR did not tally with estimates from Southern hybridization, we used a high-throughput sequencing approach to complement TAIL-PCR. Although most of our lines harbor multiple *Ds* integrations, the flanking sequence information can be found elsewhere (http://fishtrap.warwick.ac.uk/) and can be used to easily breed the line to single insert.

### Reporter expression profiling of *DsDELGT4* integrations

The 642 lines that were positive for reporter expression exhibited a dynamic and wide range of expression patterns. For the first 24 hr after fertilization, we observed different intensities of GFP expression, typically in the central nervous system (CNS) or ubiquitous distribution throughout the embryo (Figure S2 and Figure S3). Expression of mCherry was usually weak and ubiquitous, and we rarely observed strong or restricted Cherry expression at early stages, unless there was maternal expression (Figure S4 and Figure S5). From 2 d after fertilization onwards, we observed more lines with both reporters expressed in the CNS, especially strong in the eyes and specific sub-domains in the developing brain, as well as in other cell type-specific patterns, such as expression in the branchial arches, fins, otoliths, heart tube, notochord, hatching gland, and various muscle cell types (Figure S3 and Figure S5). From 3 d after fertilization onwards, reporter expression was observed in more organs and tissues, such as the liver, pancreas, pronephric ducts, intestine, olfactory placodes, cranial cartilages, and blood vessels.

We observed mCherry expression in more than 70 lines at 24 hr after fertilization (Figure S4), and we even observed maternal mCherry expression in one-cell stage embryos from 59 lines. However, the level of mCherry expression in some lines is low and sometimes difficult to observe by eye alone. This might be due to a combination of three factors. First, mCherry expression levels are controlled by endogenous target gene promoters that might be expressed at low levels during the early stages when we assessed the reporters. Second, successful protein trapping results in a range of different fusion proteins, some of which might interfere with fluorescent activity of mCherry or may be out of frame. Finally, replacement of the 3′ end of the endogenous mRNA by mCherry might result in the absence of RNA stabilizing sequences.

**Figure 2:**
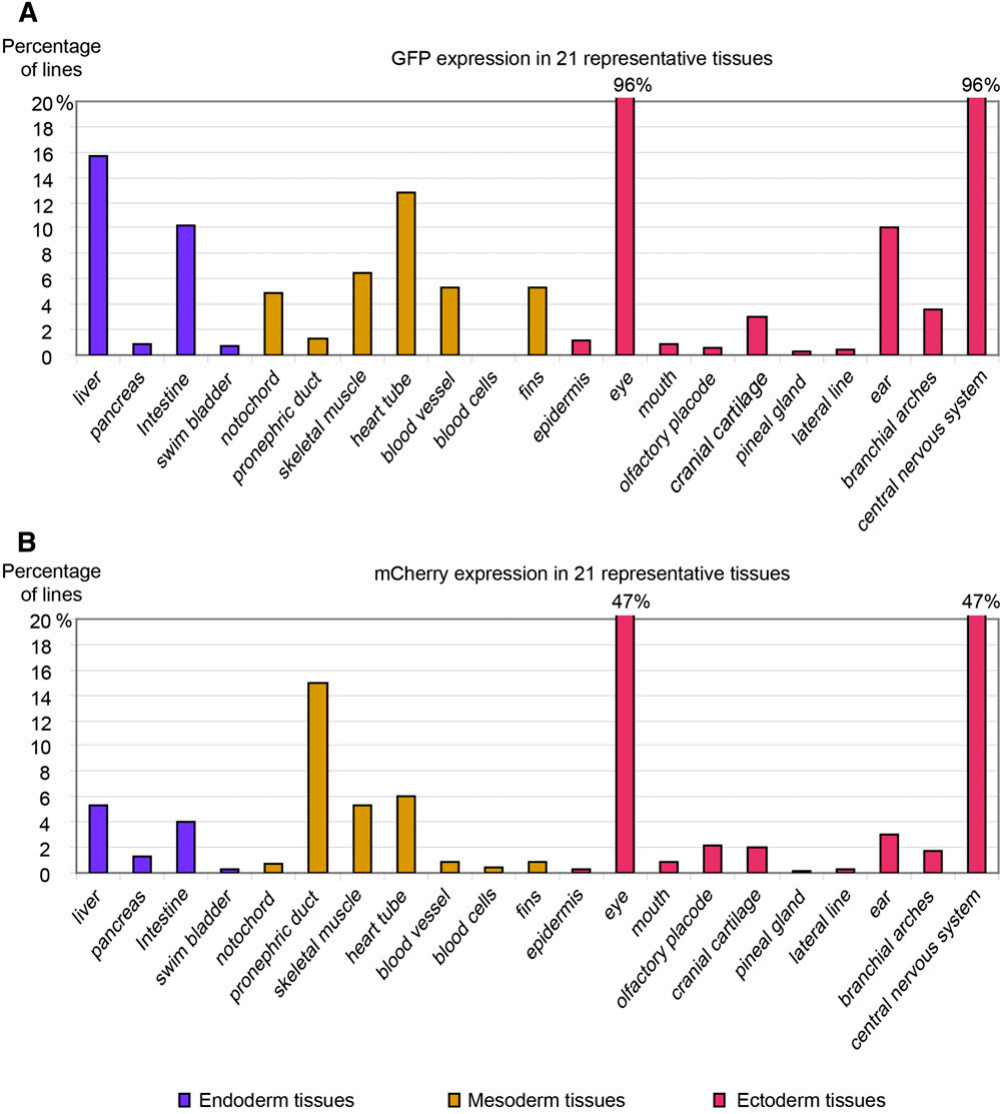
Reporter expression in different tissues. Graphs in (A) and (B) show percentage of reporter positive lines (n = 642) with enhancer trap reporter GFP expression (A) and protein trap reporter mCherry expression (B) in 21 tissues/organs derived from the three germ layers.

Many lines exhibited strong expression of both reporter**s** in the CNS, but we also identified brighter expression in more specific regions, such as the mid-hindbrain boundary (MHB), forebrain-midbrain boundary (FMB), and rhombomeres of the hindbrain (Figure S2 and Figure S4). In fact, 304 protein trap lines and 616 enhancer trap lines showed expression in the CNS and eyes, although we rarely see eye expression alone, but we do observe it in combination with CNS expression (Figure 2, A and B). For enhancer traps, we used a short promoter from the *gfap* gene, which encodes an intermediate filament protein expressed by numerous cell types of the CNS (Eng *et al.* 2000; Kuzmanovic *et al.* 2003). This may partially explain the high proportion of lines with GFP expression in the CNS. After 24 hr after fertilization, and similar to previous reports in which 30–75% of lines exhibit neuronal expression (Clark *et al.* 2011; Stoykova *et al.* 1998; Wurst *et al.* 1995), we also identified a large proportion of protein trapped lines with mCherry expression in the head and neural tube. Transgenic lines and mutants isolated from our screens can provide helpful information to further unravel the molecular hierarchies involved in the development of the CNS.

**Figure 3:**
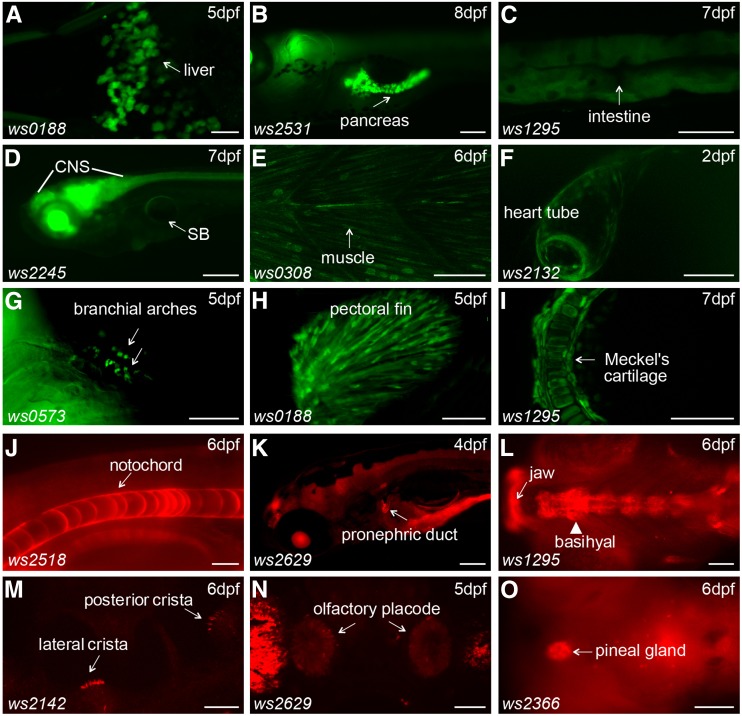
Reporter expression in various cell types. (A–I) Examples of enhancer trap reporter GFP expression patterns in the liver (A), pancreas (B), intestine (C), central nervous system (D), swim bladder (D), muscle cells (E), heart tube (F), branchial arches (G), fins (H), and cranial cartilage (I). (J–O) Examples of protein trap reporter mCherry expression patterns in notochord (J), anterior pronephric duct (K), mouth (L), inner ear (M), olfactory placode (N), and pineal gland (O). Ventral views are shown in (A) and (G). Lateral views are shown in (B–F), (H), (J), (K), and (M), with anterior to the left. Dorsal views are shown in (I), (L), (N), and (O). CNS, central nervous system; SB, swim bladder. Scale bars represent 100 µm in (B), 200 µm in (D), 20 µm in (J) and (M), and 50 µm in other images.

**Figure 4:**
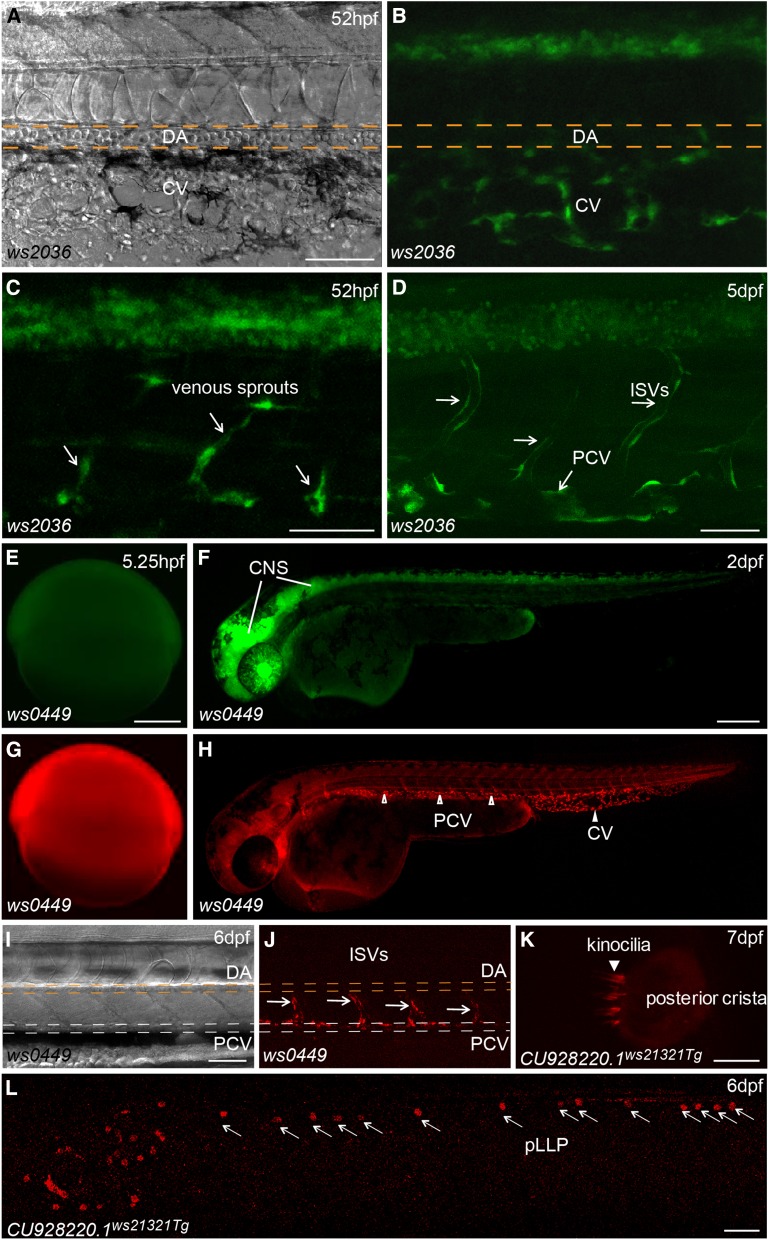
Tissue-specific expression patterns may reveal activities of trapped loci. (A–D) Bright field (A) and fluorescent images of *Tg(DsDELGT4)ws2036* embryos at 52 hr after fertilization (A–C), and 5 d after fertilization (D). Arrows show GFP expression in venous sprouts from posterior cardinal vein (C) and inter-segmental veins (ISVs) (D). (E–J) Bright field (I) and fluorescent images of *Tg(DsDELGT4)ws0449* embryos at 5.25 hr after fertilization (E, G), 2 d after fertilization (F, H), and 6 d after fertilization (I, J). GFP expresses in CNS and mCherry expresses in posterior cardinal vein (PCV), caudal vein (CV) (arrowheads in H), and intersegmental veins (arrows in J), but not in dorsal aorta (DA in J). (K, L) Fluorescent images of a *CU928220.1^ws21321Tg^* embryo with mCherry expression in the inner ear of a larva 7 d after fertilization (K, arrowhead) and in neuromasts of the posterior lateral line primordium (pLLP) (L, arrows) at 6 d after fertilization. Scale bars, 50 µm in (A, C, D, E, I), 250 µm in (F) and (L), and 10 µm in (K).

Besides the CNS, we also found reporter expression in various tissues/organs derived from all three germ layers of zebrafish embryos (Figure 2, A and B and Table S1, Table S2, Table S3, Table S4, Table S5, Table S6, Table S7, Table S8, Table S9, Table S10, Table S11, Table S12, Table S13, Table S14, Table S15, Table S16, Table S17, Table S18, Table S19), including expression in the liver (Figure 3A), pancreas (Figure 3B), intestine (Figure 3C), swim bladder (Figure 3D), notochord (Figure 3J), pronephric ducts (Figure 3K), various muscles (Figure 3E), the heart tube (Figure 3F), branchial arches (Figure 3G), fins (Figure 3H), jaw (Figure 3L), olfactory placodes (Figure 3N), cranial cartilages (Figure 3I), otoliths (Figure 3M) and the pineal gland (Figure 3O). Many lines show diverse expression patterns at different developmental stages, indicating dynamic expression of the tagged genes and/or multiple insertions in the founders. Tissue-specific expression patterns are usually a direct readout of protein activity, indicating where the protein product functions. For example, the *Tg(DsDELGT4)ws2036* line contains an insertion 333.13 kb upstream of the translation start site of the zebrafish *nr2f2* (Coup-TFII) gene. The transcription factor COUP-TFII functions as a determinant for venous cell fate specification and promotes venous identity by inhibiting expression of arterial-specific genes (You *et al.* 2005). Interestingly, the enhancer trap reporter GFP in *Tg(DsDELGT4)ws2036* embryos is expressed strongly in the vein and venous sprouts at 52 hr after fertilization (Figure 4, A–C). Later, at 5 d after fertilization, GFP expression is restricted to the posterior cardinal vein (PCV) and intersegmental veins, but not in the dorsal aorta and intersegmental arteries (Figure 4D).

We found that 338 lines showed both GFP and mCherry expression during the first 7 d of development. For example, the *Tg(DsDELGT4)ws0449* line shows ubiquitous GFP and mCherry expression during gastrulation (Figure 4, E and G); however, GFP expression is gradually restricted to the CNS (Figure 4F), whereas mCherry expression becomes stronger in the vasculature and is later detected exclusively in venous cells (Figure 4, H–J). The spatio-temporal distribution of GFP does not necessarily overlap with dynamic mCherry expression in the same embryo, suggesting the presence of multiple trapping insertions. Moreover, the same protein trap insertion that causes mCherry expression does not always show GFP expression in the same cells. In certain cell types, the short GFAP promoter sequences may not capture the enhancer of the gene where it resides, perhaps due to the distance or genomic architecture, such that the transcription initiation complex does not form. We also identified 59 lines with maternal GFP and/or mCherry expression in one-cell stage embryos (data not shown), indicating possible maternal functions for the tagged genes.

Many lines in the collection likely contain multiple integrations. In the case of single insert lines, we find that insertions falling within an intron or exon in the same direction of the gene can give rise to mCherry expression [*e.g.*, *Tg(DsDELGT4)ws1894* and *Tg(DsDELGT4)ws0310*]. In contrast, GFP expression can result from insertions in intergenic regions [*e.g.*, *Tg(DsDELGT4)ws2027*] and introns in sense [*e.g.*, *Tg(DsDELGT4)ws1309*] or antisense directions [*e.g.*, *Tg(DsDELGT4)ws1782*].

Reporter expression can provide useful information to predict the role of tagged novel genes. The *Tg(DsDELGT4)ws21321* line has an insertion in intron 1 of a novel gene *CU928220.1* (full cDNA sequence predicted from EST sequences EE203339, EE302153, and Ensembl predicted transcript ENSDART00000112096), which shows sequence similarity to neuron-derived neurotrophic factor (NDNF). The ws21321 protein trap reporter is expressed exclusively in neuromasts of the lateral line system, which is important for schooling behavior, predation, and orientation of fish, and it is regarded as a model to study the mechanisms of hearing (Chitnis *et al.* 2012; Froehlicher *et al.* 2009). We found mCherry-expressing cells were deposited along the length of the embryo, from the otic vesicle to the tip of the tail, consistent with migration of the posterior lateral line primordia (pLLP) (Figure 4L). When we looked carefully into the posterior crista of the inner ear, enhanced mCherry expression was observed in ciliary bundles (Figure 4K). The expression pattern of mCherry fusion protein indicates a possible role for the *CU928220.1* gene in the development and maintenance of neuromasts, similar to some brain-derived neurotrophic factors (Germana *et al.* 2010). This demonstrates that our *Ds* integrations show the potential for novel gene and novel transcript discovery. Thus, the collection of tissue-specific reporter lines from our DsDELGT4 screen provides an opportunity to identify novel signaling pathways and factors involved in differentiation of various tissues (*e.g.*, vein *vs.* artery development) and constitutes a useful tool for studying organogenesis.

### Annotation of insertions

**Figure 5:**
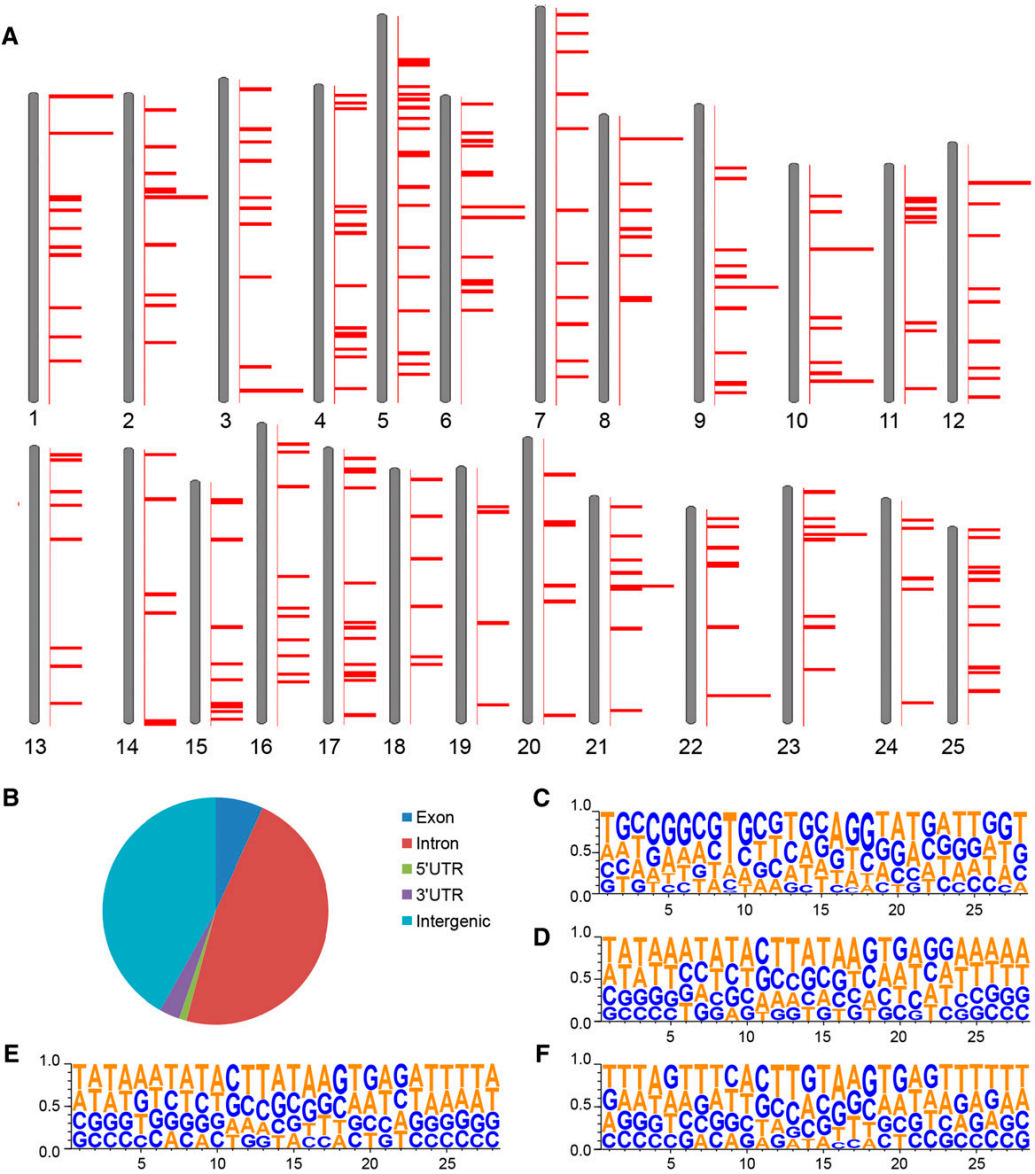
Mapping of *DsDELGT4* integrations to zebrafish chromosomes. Two hundred seventy-seven integrations sites (identified by TAIL-PCR from 283 founder lines) were used for this analysis. (A) Relative positions of integration sites on each chromosome according to zebrafish reference genome Zv9. (B) Distribution of insertions in specific regions of annotated genes (n = 277 insertions). (C–F) Alignment of 28 nucleotides around the integration sites located in intronic (C; n = 131), exonic (D; n = 30), or intergenic regions (E; n = 116) compared to all mapped *Ds* insertion sites (F). The eight nucleotides duplicated at integration sites are at nucleotide positions 11 to 18.

Dynamic reporter expression is usually associated with protein functions of tagged genes, so it is important to identify the corresponding insertions. However, most of the reporter lines have complex protein trap and/or enhancer trap expression patterns due to the tagging of many genes by multiple *Ds* insertions, making it difficult to assign each specific expression pattern to a particular insertion by a single method. We used TAIL-PCR to recover the flanking sequences of the *Ds* insertions, performed 5′ rapid amplification of cDNA ends (5′ RACE) to identify the trapped genes, and we also utilized a high-throughput sequencing method in cases where both TAIL-PCR and RACE failed. We mapped 467 sequences from 310 reporter-positive lines by TAIL-PCR and RACE and found them to be distributed across all 25 chromosomes with no obvious bias or recognizable hotspots (based on Zv9; Figure 5A). We identified 277 unique *Ds* insertion sites. Fish with 212 of the insertions were successfully propagated and sperm has been cryopreserved. Approximately 42% of the 277 insertions map to intergenic regions, but the majority were found in introns (47%), exons (7%), or UTRs of known genes (4%), some of which potentially lead to protein trap events (Figure 5B).

Ds integrations occurred in a wide range of gene types (Table 2), including translational regulators [*e.g.*, msi2 in *Tg(DsDELGT4)ws1208*], signaling receptors [*e.g.*, ptprub in *Tg(DsDELGT4)ws0148*], helicases [*e.g.*, dhx37 in *Tg(DsDELGT4)ws0977*], and microtubule motors [*e.g.*, dnah7l in *Tg(DsDELGT4)ws2293*]. Although we observed enrichment of some gene ontology terms (*e.g.*, WD40 repeat), these were not statistically significant on correction for multiple testing (Benjamini p-value 0.17; Table S20). Alignment of a 28-nucleotide sequence around the 277 mapped integration sites did not show any obvious consensus sequence or preferential site for the *DsDELGT4* integrations (Figure 5, C–F). We found an additional 54 flanking sequences that could not be mapped unambiguously to chromosomes. These insertions possibly fall in repetitive regions of the zebrafish genome. As the molecular characterization of the integrations progresses, and as the annotation of the zebrafish genome improves further, we expect that more insertion sites will be resolved and new genes and transcripts may be discovered (http://fishtrap.warwick.ac.uk/).

**Table 2:** List of mutants for some novel genes

Line ID	Chromosome	Start	End	Affected Gene	Orientation	Location	Reference Transcript
ws0148	16	37705719	37705727	*protein tyrosine phosphatase*, *receptor type*, *U*, *b (ptprub)*	Antisense strand	Intron 6	ENSDART00000078970
ws0449	17	52113334	52113341	*peroxidasin (pxdn)*	Sense strand	Intron	LOC570177
ws0548	25	15633196	15633204	*t-complex 11 (mouse)-like 1 (tcp11l1)*	Antisense strand	Exon 5	ENSDART00000045659
ws0977	8	4765207	4765215	*dhx37*	Antisense strand	Intron 4	ENSDART00000127153
ws0977	2	20293551	20293559	*amylo-1*, *6-glucosidase*, *4-alpha-glucanotransferase a (agla)*	Sense strand	Intron 32	ENSDART00000007766
ws1208	10	39111748	39111756	*musashi homolog 2a (msi2a)*	Sense strand	Intron 13	ENSDART00000099473
ws1342	15	38821797	38821805	*CR388129.2*	Antisense strand	Exon	ENSDART00000122265
ws1357	6	36507540	36507548	*optic atrophy 1 (OPA1)*	Antisense strand	Intron 27	ENSDART00000104256
ws2110	13	42793923	42793931	*CDC42 effector protein (Rho GTPase binding) 3 (cdc42ep3)*	Sense strand	Intron 2	ENSDART00000074707
ws2132	17	26415130	26415137	*CU928220.1*	Sense strand	Intron	ENSDART00000112096
ws2293	1	20842629	20842637	*dynein*, *axonemal*, *heavy polypeptide 7 like (dnah7l)*	Antisense strand	Exon 2	ENSDART00000054388
ws2293	20	28937519	28937527	*si:dkey-11e23.5*	Antisense strand	Intron	ENSDART00000046042
ws2608	2	51165	51173	*CABZ01090951.1*	Antisense strand	Exon	ENSDART00000066767
ws2608	4	29196311	29196319	*BX323023.1*	Sense strand	Exon	ENSDART00000143145

The insertion positions are according to ZV9 genome assembly. For an effective protein trap, the orientation of the gene and insert should be the same if the insertion falls into introns. The exact exon/intron location of insertions is only given if the transcript is complete.

### Functions of tagged genes

**Figure 6:**
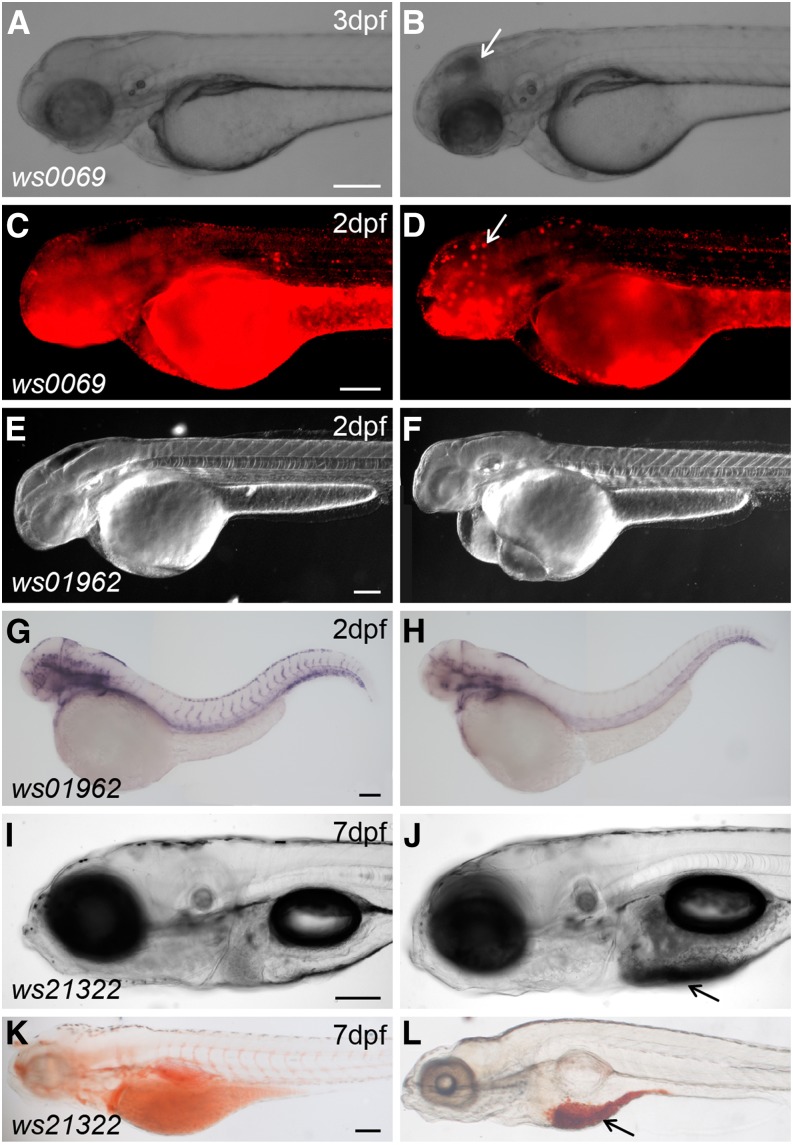
Representative mutant phenotypes in *DsDELGT4* insertion lines. The *Tg(DsDELGT4)ws0069* mutant shows cell death in the brain (B) compared to siblings (A) (bright field images). (C, D) Acridine Orange labeling to show cell death in the brain of mutant embryos (D) compared to siblings (C). White arrows in (B) and (D) show cell death in mutants. DIC images of *Tg(DsDELGT4)ws01962* mutants showing smaller head and edema (F) compared to siblings (E). (G) and (H) *In situ* hybridization staining results with a probe to detect the endothelial marker *flk1*. Expression of *flk1* is dramatically reduced in mutants (H) compared to siblings (G). *Tg(DsDELGT4)ws21322* mutants exhibit fat metabolism defects (J) compared to siblings (I). (K) and (L) Oil red O staining in sibling (K) and mutant (L) embryos. Nonmetabolized fat is observed below the swim bladder in mutant embryos (arrows in J and L). Scale bars, 100 µm. Lateral views shown.

By phenotypic analysis of 283 lines, we identified 21 phenotypes (7.5%) from sibling matings of heterozygous carriers. For instance, line *Tg(DsDELGT4)ws0069* shows cell death in the brain (Figure 6, A–D), *Tg(DsDELGT4)ws01962* shows defects in vasculature development (Figure 6, E–H), and *Tg(DsDELGT4)ws21322* shows aberrant yolk fat metabolism (Figure 6, I–L). However, the overall percentage of phenotypic mutations is likely much larger, because we only concentrated on obvious or lethal phenotypes up to 7 d after fertilization and did not score subtle phenotypes, those requiring comparisons of marker gene expression, or other specific phenotypes (such as behavior or physiology). Based on the number of integrations occurring within the first exon or intron, we estimate that 12% of insertions are likely mutagenic. It is expected that further exploration of this collection should yield higher numbers of affected loci. Further characterization is required to determine the precise molecular mechanisms by which these integrations disrupt gene function.

**Figure 7:**
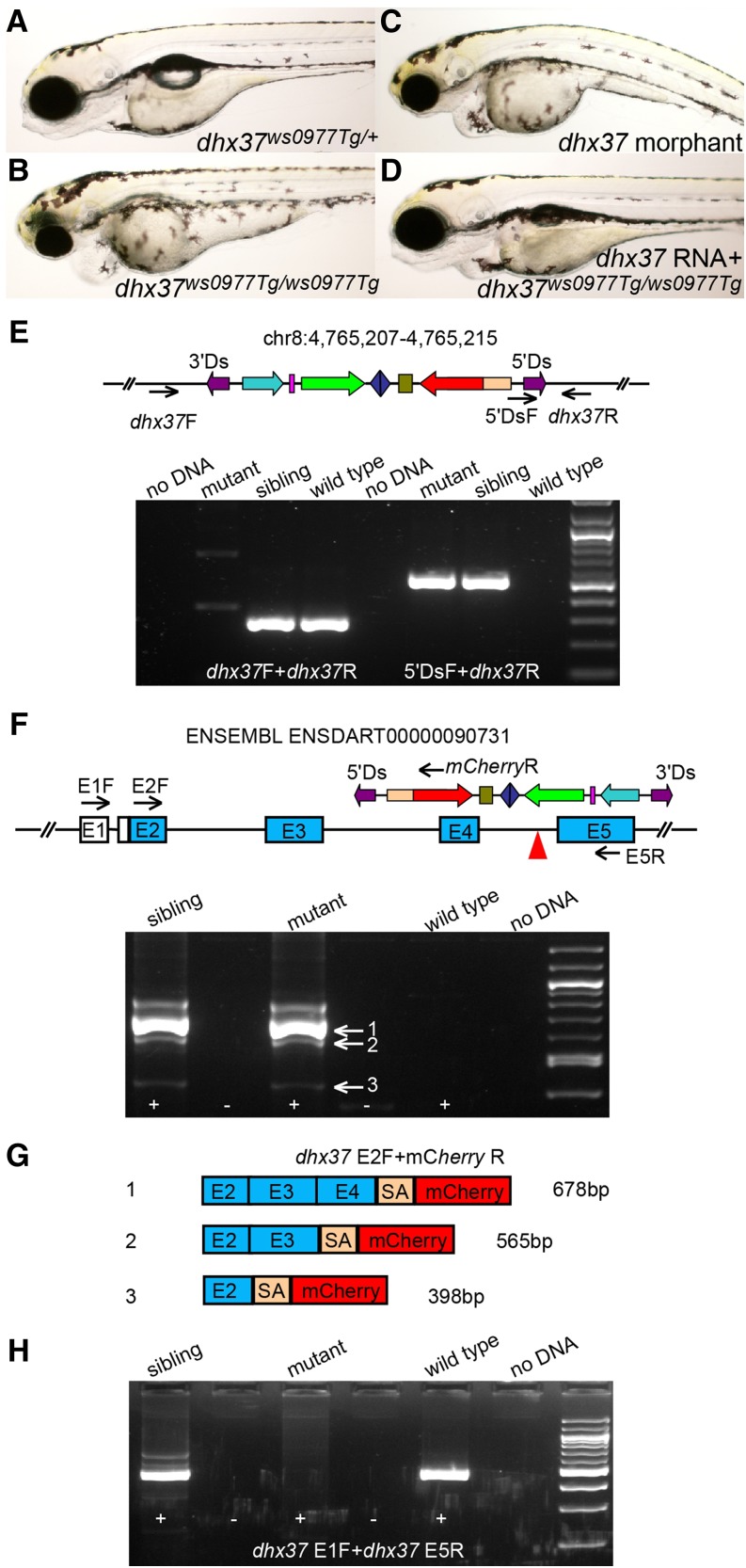
Protein trap-induced mutant phenotype affecting the *dhx37* gene. (A–D) Bright field images of *dhx37^ws0977Tg/+^* sibling (A), homozygous *dhx37^ws0977Tg/ws0977Tg^* mutant (B), *dhx37* ATG morpholino-injected (C), or full-length *dhx37* RNA-injected homozygous mutant embryo (D). (E) The *Ds* insertion in chr8:4765207-4765215 is linked to the mutant phenotype. Black arrows indicate the position of primers used for genotyping PCRs. (F–H) RT-PCR results from homozygous *dhx37^ws0977Tg/ws0977Tg^* embryos show that the *bcl2* splice acceptor sequence trapped endogenous upstream splice donor sites in the *dhx37* gene, producing multiple fusion transcripts (F, G), leading to the disruption of wild-type transcripts (H). (G) The main fusion products, 1 and 2, are out of frame with respect to the reading sequence. Black arrows in the schematic in (F) indicate the positions of primers used for RT-PCR. No evidence of wild-type transcripts was found, and only mutant fusion transcripts were detected. The fusion transcripts always have a 71-bp linker sequence (SA) originated from *bcl2* splice acceptor site left between upstream exons and the *mCherry* reporter sequence. The major fusion transcripts 1 and 2 are out of frame for *mCherry* coding sequences (G).

We have also identified potential protein trap events in many lines based on *Ds* integration locations identified from TAIL-PCR or RACE results. These include integrations in novel genes as well as known genes whose functions have not been described previously (Table 2). Although not every protein trap event gives rise to mCherry expression, depending on the strength of expression and whether *mCherry* is fused in frame with upstream endogenous exons, these protein trap lines allow us to generate mutants and assess the functions of the trapped loci. We describe the characterization of the *dhx37^ws0977Tg^* insertion as a representative example. The *Tg(DsDELGT4)ws0977* line harbors an insertion in intron 4 of the DEAH (Asp-Glu-Ala-His) box polypeptide 37 (*dhx37*) gene. The *dhx37* gene encodes a putative RNA helicase. RNA helicases are usually involved in various aspects of RNA metabolism and play roles in differentiation and carcinogenesis (Abdelhaleem *et al.* 2003; Alli *et al.* 2006). F2 adult carriers with the *dhx37^ws0977Tg^* insertion were inter-crossed to generate *dhx37^ws0977Tg^* homozygotes. Mutant embryos exhibited edema and overall reduction in the anterior brain, as well as a shortened body axis (Figure 7, A and B). These developmental defects can be phenocopied by injecting *dhx37* ATG morpholinos (35%, n = 62; Figure 7C) and splice morpholinos toward the intron 4/exon 5 acceptor site of *dhx37* (28%, n = 60) into one-cell stage wild-type embryos. By co-injecting wild-type *dhx37* RNA, mutant phenotypes can be rescued to wild-type morphology (Figure 7D). The flanking genomic sequence of the *Ds* insertion was mapped to chromosome 8, and the orientation of *mCherry* was found to be the same as that of the *dhx37* gene. Single PCR products containing the 5′ end *Ds* sequence and adjacent flanking genomic sequence were amplified in mutants and siblings, but not in wild-type embryos. By contrast, a pair of primers spanning the integration site can amplify robustly in sibling and wild-type embryos, but not in mutants. Taken together, these results show that the *Ds* transposon integration within the *dhx37* gene is responsible for the mutant phenotype observed in *dhx37^ws0977Tg^* homozygotes (Figure 7E).

To determine whether the *bcl2* splice acceptor in the *Ds* insertion trapped the *dhx37* transcripts, we prepared total RNA from *dhx37* mutant, sibling, and wild-type embryos at 3 d after fertilization and performed reverse-transcription PCR (RT-PCR) using a primer in the first coding exon (exon 2) of the *dhx37* gene and a primer in the *mCherry* coding sequence. Multiple fusion products were detected in mutants and siblings, indicating that the *bcl2* splice acceptor trapped the dhx37 transcript (Figure 7F). Sequencing the fusion products showed that the *bcl2* splice acceptor from this insertion not only trapped the most adjacent donor site but also trapped all the upstream splice donor sites of the same gene (Figure 7G). The distance between the splice donor and acceptor sites may influence trapping efficiency (Figure 7, F and G). The major fusion products are out-of-frame for *mCherry* coding sequence (Figure 7G), which may explain why mCherry reporter expression is barely detected in *dhx37* mutants and siblings. In addition, when we used a primer targeting the 5′ noncoding exon (exon 1) of the *dhx37* gene and another primer toward sequences in exon 5 (the exon immediately downstream of the *Ds* insertion) by standard RT-PCR, we could not amplify any PCR products from mutants embryos and only mutant fusion transcripts were detected. This suggests that the protein trap insertion in *Tg(DsDELGT4)ws0977* disrupted normal splicing.

### Database of *Ds* transposon integrations

**Figure 8:**
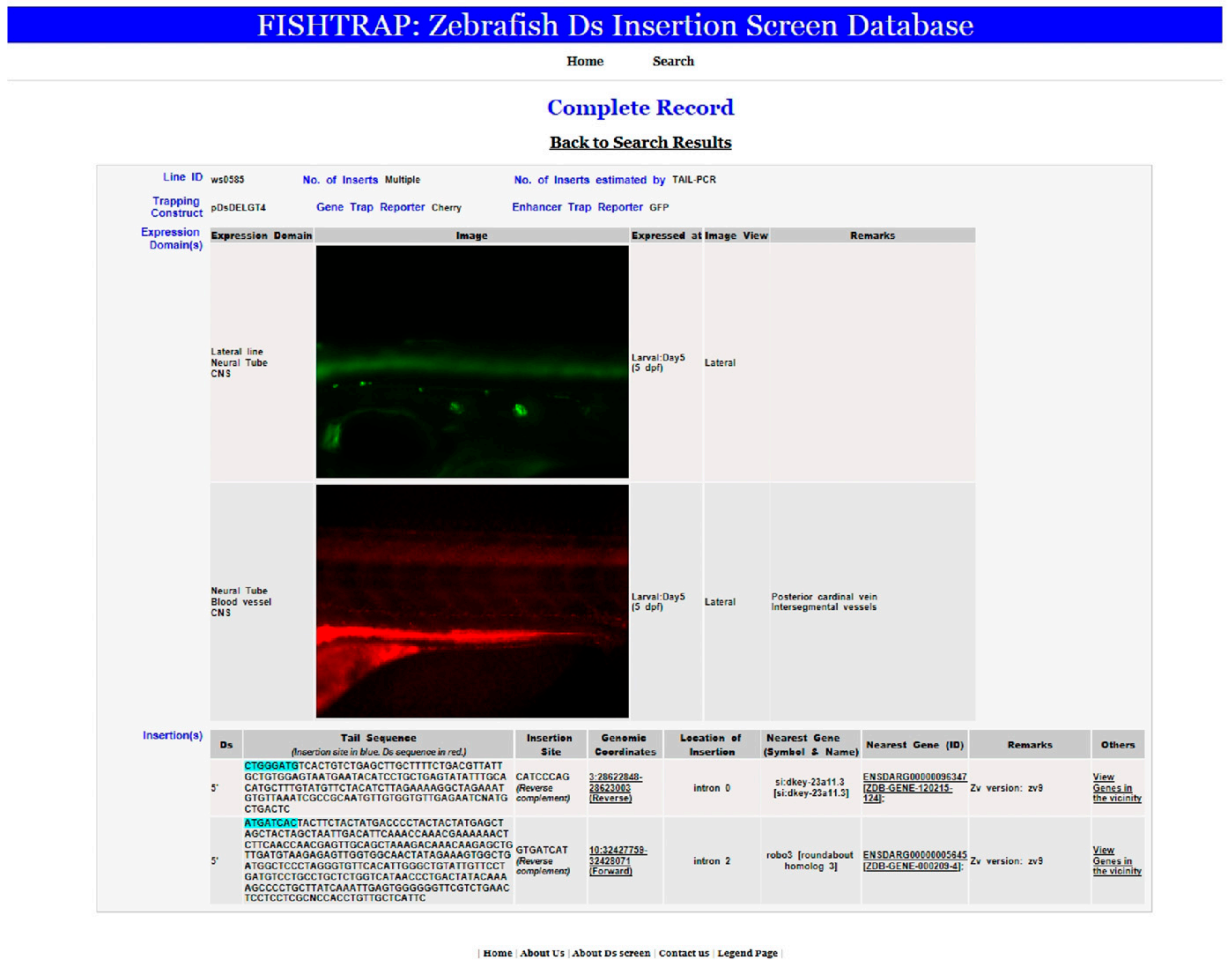
The FISHTRAP database of *DsDELGT4* line expression and molecular data. This web-based interface stores reporter expression and flanking sequence information for the *DsDELGT4* lines and can be searched by multiple parameters: line number, expression domain, developmental stage, and gene name. An example of a record for *Tg(DsDELGT4)ws0585* is shown as a screenshot. *Ds* integration sites were mapped to the zebrafish reference genome assembly (Zv9). Reporter expression patterns are displayed in the sequence of developmental stages and anatomical structures are indicated.

Our *Ds* integrations disrupt gene functions in the manner in which a fusion protein may be expected. It provides a rich resource of mutants and tissue-specific transgenic reporter lines, complementing other large-scale screens to facilitate the identification and functional study of known as well as novel genes and transcripts. We developed a web-based interface to store and report the expression and molecular analysis of *DsDELGT4* lines (http://fishtrap.warwick.ac.uk/). The website provides information that includes reporter expression patterns, integration sites, and flanking sequence data. As more integration sites and trapped genes are identified, more data will be made available. For data inquiry, the interface can be simply searched by four parameters: line number, expression domains, developmental stage, and gene name. In the advanced search function, the chromosome, scaffold, and integration site are additional fields for inquiry. The system can also show records that match multiple or any of the parameters. Figure 8 shows an example of the record for line *Tg(DsDELGT4)ws0585*. Cryopreserved sperm from the *Ds* integration lines are being deposited with the European Zebrafish Resource Centre (http://www.ezrc.kit.edu/) to be made available to the broader research community.

### Applications of the *DsDELGT4* insertion collection

**Figure 9:**
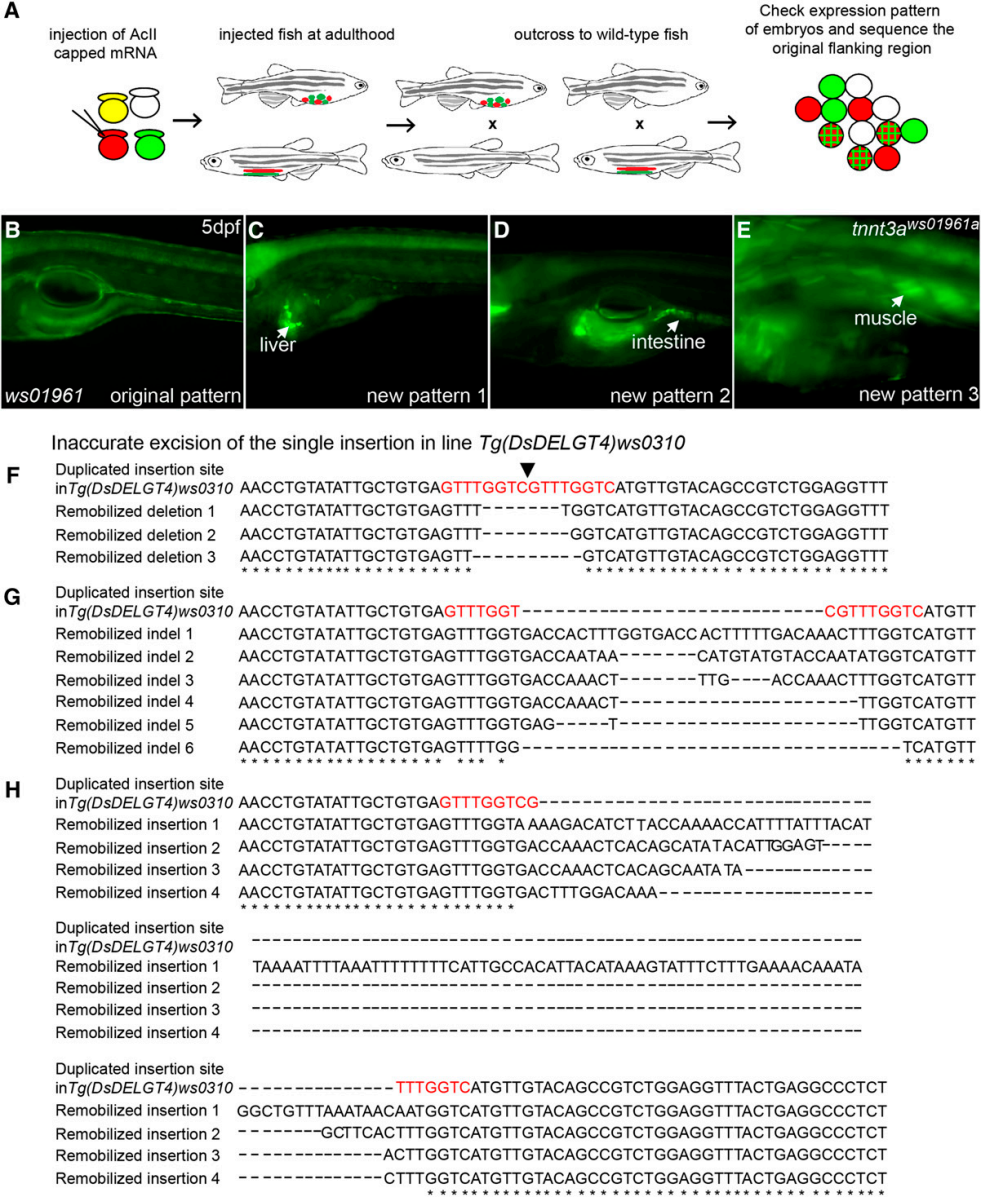
Remobilization of *DsDELGT4* leads to imprecise excision and generates integrations with new reporter expression patterns. (A) Schematic representation of the remobilization experiment. Ac transposase mRNA was injected into one-cell stage embryos that show reporter expression. The injected embryos are raised to adulthood and mated with wild-type fish. Their progeny were examined for sequences flanking the original integration site and screened for new reporter expression patterns. (B–E) Remobilization of *Ds* in embryos of the single insertion line *Tg(DsDELGT4)ws01961* (antisense strand, chr1:20205236-20205244) generates novel expression patterns (C–E) distinct from the original expression pattern (B). (F–H) Imprecise excision of *DsDELGT4* by Ac-mediated remobilization causes mutations at the original integration site (location of the original insert is shown by black arrowhead in F), including deletions (F), indels (G), and insertions (H). Genomic DNA for sequence analysis (F–H) was obtained from the embryos of *Tg(DsDELGT4)ws0310* (sense strand, chr21:17731630–17731722) and collected 24 hr after injection of Ac transposase mRNA.

The *DsDELGT4* transposon system provides opportunities to modify the integrations for various purposes. Re-introducing the Ac transposase will lead to excision of existing *DsDELGT4* insertions. A precise *Ds* excision leaves an 8-bp duplication footprint (Saedler and Nevers 1985). However, we observed that in 43% of re-mobilizations, inaccurate excisions occurred and led to various mutations in the original locus, including local deletions, insertions, and indels (Figure 9, A, F–H). This feature can therefore enable targeted genetic manipulation at each integration locus. With aberrant excision, it should be possible to easily generate multiple alleles for the transposon-tagged loci. Similarly, on excision of mutant-phenotype-causing protein trap integrations, we can expect rescue of the mutant phenotypes in subsequent generations. At the same time, re-integration of *DsDELGT4* into new loci of the genome can produce new expression patterns that are distinct from the original *Ds* line, indicating novel protein trap or enhancer trap events (Figure 9, B–E). By using such a strategy, we acquired a protein trap line *tnnt3a^ws01961aTg^* with the *Ds* insertion in exon 6 of the *troponin T3a* gene (chr25:32,256,224-32,256,231, sense strand) derived from remobilization of the single insertion (chr1:20205236-20205244, antisense strand) in the *Tg(DsDELGT4)ws01961* line (Figure 9, B and E). It was reported that *tnnt3a* gene is expressed in fast-twitch muscles, head muscles, pectoral fin muscles, and hypaxial muscles from 18 hr after fertilization in zebrafish (Ferrante *et al.* 2011). Our *tnnt3a^ws01961aTg^* embryos also show GFP and mCherry expression in these cells, consistent with the published reports. Dominant mutations in *TNNT3* cause distal arthrogryposis (DA) disorders in humans (Sung *et al.* 2003), and depletion of *tnnt3a* in zebrafish via morpholinos blocks normal myofibrillogenesis (Ferrante *et al.* 2011). The *tnnt3a^ws01961aTg^* line generated by re-integration of *DsDELGT4* insertion may provide a useful model to study the function of *tnnt3a* and to understand the basis of DA syndromes in humans. Thus, iterative remobilizations of the *DsDELGT4* integrations can offer near-infinite possibilities to visualize the expressed genome, to provide information regarding the functions of trapped genes, and to specifically modify the genome in a directed manner.

## Discussion

Our pDsDELGT4 is a multifunctional vector whose integration simultaneously enables visualization of the expressed genome and its mutagenesis. The *Ds* insertions trap enhancers of endogenous genes and reveal their expression with GFP tagging. When protein traps happen with reporter fusions in frame, mCherry expression exhibits endogenous protein expression information to some extent. In cases where mCherry fusion occurs out of frame, the expression of fusion products can be unmasked by *in situ* hybridization with antisense *mCherry* probes. Depending on the insert location, mCherry-tagged fusion proteins may terminate at different positions along the peptide. If fusion happens at the N-terminus, then it is more likely to produce functional protein-null alleles, providing useful tools to analyze the function of the gene product. If fusion happens at the C-terminus, then it is more likely to produce proteins with partial functions. Although the expression of the protein trap reporter may not faithfully reflect the wild-type protein expression of trapped genes, the truncated fusion proteins may retain at least some information, such as protein localization, facilitating prediction of protein functions. Because our lines show reporter expression in various cell types, they may reveal multiple novel signaling pathways and factors in the development of labeled organs.

The percentage of phenotypic mutations observed was low, similar to previously reported screens (Kettleborough *et al.* 2013). There are several reasons for this. First, we only monitored morphological changes in embryos during the first week of development. The assay used was not efficient for discovering subtle phenotypes that may require in-depth investigation or defects that may arise at later stages. Second, paralog redundancy is common in the zebrafish genome (Postlethwait *et al.* 2000). Third, based on the location of the protein trap, a mutation may or may not disrupt gene function and manifest an obvious phenotype. Fourth, in some homozygous mutants, maternal contribution of the gene products may compensate for their zygotic functions; therefore, embryos from homozygous mutant females will need to be examined to assess the function of these genes. Nonetheless, we recovered some obvious mutant phenotypes by visually screening embryos from random intercrosses of F2 or F3 generation siblings (Figure 6). We have also identified many potential protein trap insertions based on the integration locations (Table 2). Therefore, it may be possible to identify more phenotypic mutations in the future.

We showed that the *Ds* collection can be used to create additional enhancer and protein trap events by remobilization of the existing *Ds* insertions. This strategy can be useful for achieving saturation mutagenesis and for obtaining more comprehensive gene and enhancer trap expression patterns. With our *Ds* cassette design, the presence of two or more *DsDELGT4* insertions in the same genome can also allow Cre-lox–mediated recombination, which can lead to defined chromosome segment inversions, translocations, or deletions, depending on the orientation and location of the lox2272 sites in *DsDELGT4* integrations. For instance, by injecting Cre recombinase RNA into embryos with two *DsDELGT4* insertions oriented in the same direction on a segment of chromosome 21, we were able to generate a 25-Mb deletion (with a deletion frequency of ∼7%; Figure S6), encompassing the nodal-related gene, *squint*. Cre-mediated recombination can potentially also be deployed to generate precise segmental inversions between *Ds* insertion sites, which, together with reporter expression as markers, could facilitate the generation of balancer chromosomes (Muller 1918).

In recent years, a number of transposon-based protein trap and enhancer trap systems have been developed and successfully used in many cells and vertebrate organisms (Boon Ng and Gong 2011; Emelyanov *et al.* 2006; Froschauer *et al.* 2012; Huang *et al.* 2010; Song *et al.* 2011). Compared to other mutagenesis strategies, transposon-based protein trap systems produce higher mutagenesis frequencies, easier identification of mutated genes, and also report endogenous gene expression patterns (Stanford *et al.* 2001). However, the number of genes studied is much less than the protein coding genes in most target genomes, partly due to the limitations of transposons currently used. *PiggyBac* targets the tetra-nucleotide sequence TTAA and all known *Tc1/mariner* transposons, including *Sleeping Beauty* (*SB*), preferentially insert at TA dinucleotides (Ivics and Izsvak 2010). The *Tol2* element does not seem to have a consensus sequence for insertion at the primary DNA sequence level, but some studies show a pronounced preference for Tol2 to integrate close to transcriptional start sites (Grabundzija *et al.* 2010), CpG islands, and DNase I hypersensitive sites (Huang *et al.* 2010). The presence of such preferences and biases is a limitation for any large-scale mutagenesis screen. In contrast, the *DsDELGT4* insertions we mapped were found distributed across all 25 zebrafish chromosomes and we did not observe any obvious sequence or genomic region preferences for *Ds* in the 277 integration sites reported here. However, a larger dataset needs to be analyzed to definitively determine if *Ds* shows integration site preferences similar to those observed with other insertional mutagenesis tools.

The *Ac/Ds* system belongs to the same *hAT* family as *Tol2*, and it has many advantageous features: high integration rates (30% in zebrafish and medaka); no obvious consensus DNA sequence for insertion targets; and no sensitivity to overproduction inhibition, unlike the *SB* transposon for which elevated concentrations of the transposase inhibit transposition (Geurts *et al.* 2003). In contrast, *Ac/Ds*-injected embryos can tolerate up to 100 pg of Ac transposase RNA without affecting the insertion rate. The *Ac/Ds* system has a large cargo capacity (A. Emelyanov and S. Parinov, unpublished observation) and functions in a wide spectrum of hosts. Most importantly, *Ds* can integrate throughout the genome and does not cause gross rearrangements around the integration sites (Boon Ng and Gong 2011; Froschauer *et al.* 2012; Emelyanov *et al.* 2006; Trinh le *et al.* 2011).

The *Ac/Ds* system has been extensively used for mutagenesis in plants such as *Arabidopsis*, rice, and barley (Chin *et al.* 1999; Kuromori *et al.* 2004; Lazarow and Lutticke 2009; Sundaresan *et al.* 1995.), but has only been applied in a few small-scale screens in human cells, zebrafish, and medaka (Boon Ng and Gong 2011; Emelyanov *et al.* 2006; Froschauer *et al.* 2012; Trinh le *et al.* 2011). So far, our *DsDELGT4* screen in zebrafish is the first large-scale attempt to use the maize *Ac/Ds* for protein traps and enhancer traps in a vertebrate model organism. To fully understand the expression and function of a protein-coding gene, usually multiple mutant alleles will be needed, so comprehensive study of all the protein-coding genes in a vertebrate genome (for example, more than 26,000 protein-coding genes in zebrafish) requires millions of mutational events, which cannot be achieved by using a single mutagen. Therefore, the different mutagens, trapping strategies, vectors and transposon systems, and genome editing can be used to complement each other to minimize biases and achieve maximal genome coverage.

In summary, with the *DsDELGT4* system, we have successfully obtained numerous tissue-specific transgenic lines and created mutants affecting both known and novel genes. Our results in zebrafish suggest that *Ds* could be a useful tool to systematically modify the genomes of other vertebrates as well. This may be especially useful for medaka because *Tol2* is not suitable for transgenesis in this animal due to its natural inhibition in the host. Our collection of *DsDELGT4* transposon insertion lines has the potential to advance our knowledge of the biological basis for vertebrate development and human diseases.

## Supplementary Material

Supporting Information
