# Supplementary material for: A Multifunctional Mutagenesis System for Analysis of Gene Function in Zebrafish
Source: G3 (Bethesda). 2015 Apr 2;5(6):1283–99. doi: 10.1534/g3.114.015842 (PMC4478556; doi:10.1534/g3.114.015842)
Supplement: Supporting Information [file supp_5_6_1283__index.html]

A Multifunctional Mutagenesis System for Analysis of Gene Function in Zebrafish — A Multifunctional Mutagenesis System for Analysis of Gene Function in Zebrafish — Supporting Information 

# A Multifunctional Mutagenesis System for Analysis of Gene Function in Zebrafish

## Supporting Information for Quach *et al.*, 2015

**Files in this Data Supplement:**

- Supporting Information - Figures S1-S6 and Tables S1-S20 (PDF, 906 KB)
- Figure S1 - Number of inserts per line. (PDF, 167 KB)
- Figure S2 - The spatial-temporal distribution of enhancer trap reporter expression. (PDF, 520 KB)
- Figure S3 - Dynamic expression of the enhancer trap reporter in various tissues. (PDF, 189 KB)
- Figure S4 - The spatial-temporal distribution of protein trap reporter expression. (PDF, 493 KB)
- Figure S5 - Dynamic expression of the protein trap reporter in various tissues. (PDF, 188 KB)
- Figure S6 - Cre-lox mediated targeted large deletion. (PDF, 868 KB)
- Table S1 - DsDELGT4 lines with reporter expression in liver from day0 to day7. (.xlsx, 12 KB)
- Table S2 - DsDELGT4 lines with reporter expression in pancreas from day0 to day7. (.xlsx, 9 KB)
- Table S3 - DsDELGT4 lines with reporter expression in intestine from day0 to day7. (.xlsx, 10 KB)
- Table S4 - DsDELGT4 lines with reporter expression in swim bladder from day0 to day7. (.xlsx, 8 KB)
- Table S5 - DsDELGT4 lines with reporter expression notochord from day0 to day7. (.xlsx, 9 KB)
- Table S6 - DsDELGT4 lines with reporter expression in pronephric duct from day0 to day7. (.xlsx, 10 KB)
- Table S7 - DsDELGT4 lines with reporter expression in skeletal muscle cells from day0 to day7. (.xlsx, 10 KB)
- Table S8 - DsDELGT4 lines with reporter expression in heart tube from day0 to day7. (.xlsx, 11 KB)
- Table S9 - DsDELGT4 lines with reporter expression in blood vessels from day0 to day7. (.xlsx, 10 KB)
- Table S10 - DsDELGT4 lines with reporter expression in blood cells from day0 to day7. (.xlsx, 8 KB)
- Table S11 - DsDELGT4 lines with reporter expression in fin from day0 to day7. (.xlsx, 9 KB)
- Table S12 - DsDELGT4 Lines with reporter expression in epidermis from day0 to day7. (.xlsx, 8 KB)
- Table S13 - DsDELGT4 lines with reporter expression in mouth from day0 to day7. (.xlsx, 9 KB)
- Table S14 - DsDELGT4 lines with reporter expression in olfactory placode from day0 to day7. (.xlsx, 9 KB)
- Table S15 - DsDELGT4 lines with reporter expression in cranial cartilage from day0 to day7. (.xlsx, 9 KB)
- Table S16 - DsDELGT4 lines with reporter expression in pineal gland from day0 to day7. (.xlsx, 8 KB)
- Table S17 - DsDELGT4 lines with reporter expression in lateral line from day0 to day7. (.xlsx, 8 KB)
- Table S18 - DsDELGT4 lines with reporter expression in otoliths (ear) from day0 to day7. (.xlsx, 10 KB)
- Table S19 - DsDELGT4 lines with reporter expression in branchial arches from day0 to day7. (.xlsx, 9 KB)
- Table S20 - Gene ontology analysis. (.xlsx, 9 KB)
