# Supplementary material for: A Multifunctional Mutagenesis System for Analysis of Gene Function in Zebrafish
Source: G3 (Bethesda). 2015 Apr 2;5(6):1283–99. doi: 10.1534/g3.114.015842 (PMC4478556; doi:10.1534/g3.114.015842)
Supplement: Supporting Information [file supp_g3.114.015842_015842SI.pdf]

G3: Genes | Genomes | Genetics

A Multifunctional Mutagenesis System for Analysis of Gene Function in Zebrafish

Helen Ngoc Bao Quach[*,#], Shijie Tao[*,§#], Pavle Vrljicak[*,†,#], Adita Joshi[*], Hua Ruan [*], Rashmi Sukumaran[*], Gaurav K. Varshney[ǂ], Matthew C. LaFave[ǂ], The Ds screen team^, Shawn M. Burgess[ǂ], Christoph Winkler[§,**], Alexander Emelyanov[*], Sergey Parinov[*], and Karuna Sampath[§,†,1]

# These authors contributed equally

* Temasek Life Sciences Laboratory, National University of Singapore, Singapore 117604

§ Department of Biological Sciences, National University of Singapore, Singapore 117543

† Division of Biomedical Cell Biology, Warwick Medical School, Coventry, United Kingdom CV4 7AJ

ǂ National Human Genome Research Institute, National Institutes of Health, Bethesda, MD 20892-8004

** Centre for Bioimaging Sciences, National University of Singapore, Singapore 117543

^ The Ds screen team:

DBS, NUS: Joerg Renn, Yin Jun, Jan Brocher, Bernd Willems, Yao Sheng, Flora Rajaei, Ram Vinod Roy

NUS: Robyn Lim, Nur Adilah Bte Mahbob, Krishna Balasubramaniam, Choi Kwun, Ng Xiu Li

NTU: Engel Teo, Kau Jie Hung, Eirene Koh, Sim Zhi Wei; Nur Zatul Ezza Bte Zainal

Rong Li (Ngee Ann Polytechnic), Kim Wei Liang (Republic Polytechnic), Melvin Teo (Singapore Polytechnic)

NUS High School of Mathematics and Science: Kiang Teng, Phan Nop

[1]Corresponding author: Division of Biomedical Cell Biology, Warwick Medical School, University of Warwick, Coventry, United Kingdom CV4 7AJ. Email: K.Sampath@warwick.ac.uk

DOI: 10.1534/g3.114.015842

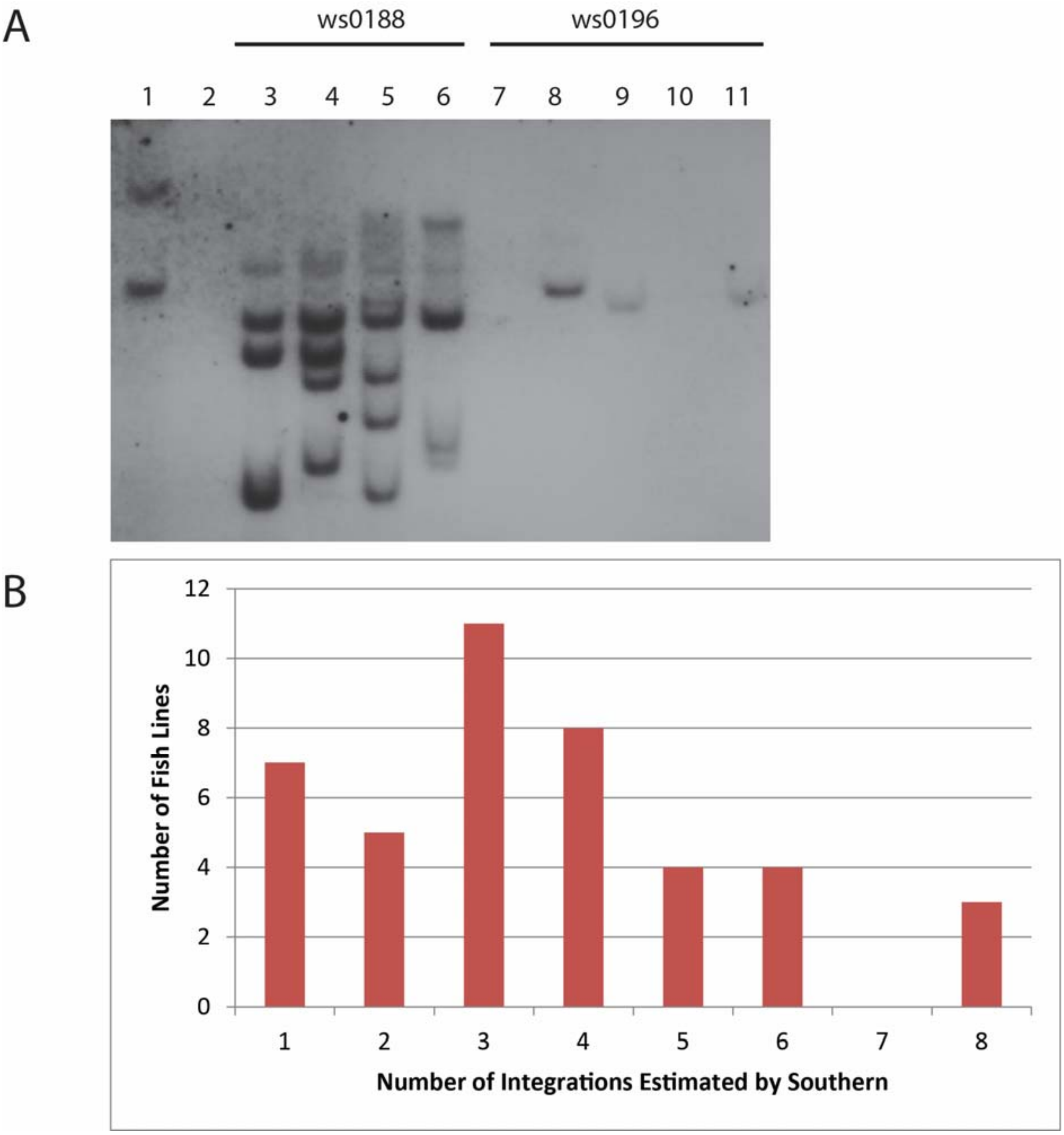

Figure S1 Number of inserts per line. Southern analysis was performed on 42 representative trapped lines to estimate the number of inserts per line. A) Representative Southern blot analysis. Lane 1: Positive control containing vector DNA and AB genomic DNA. Lane 2: Negative control containing AB genomic DNA. Lanes 3-11: Outcrosses lines *Tg(DsDELGT4)ws0188* and *Tg(DsDELGT4)ws0196* showing multiple integrations per line. B) Estimated number of inserts per line.

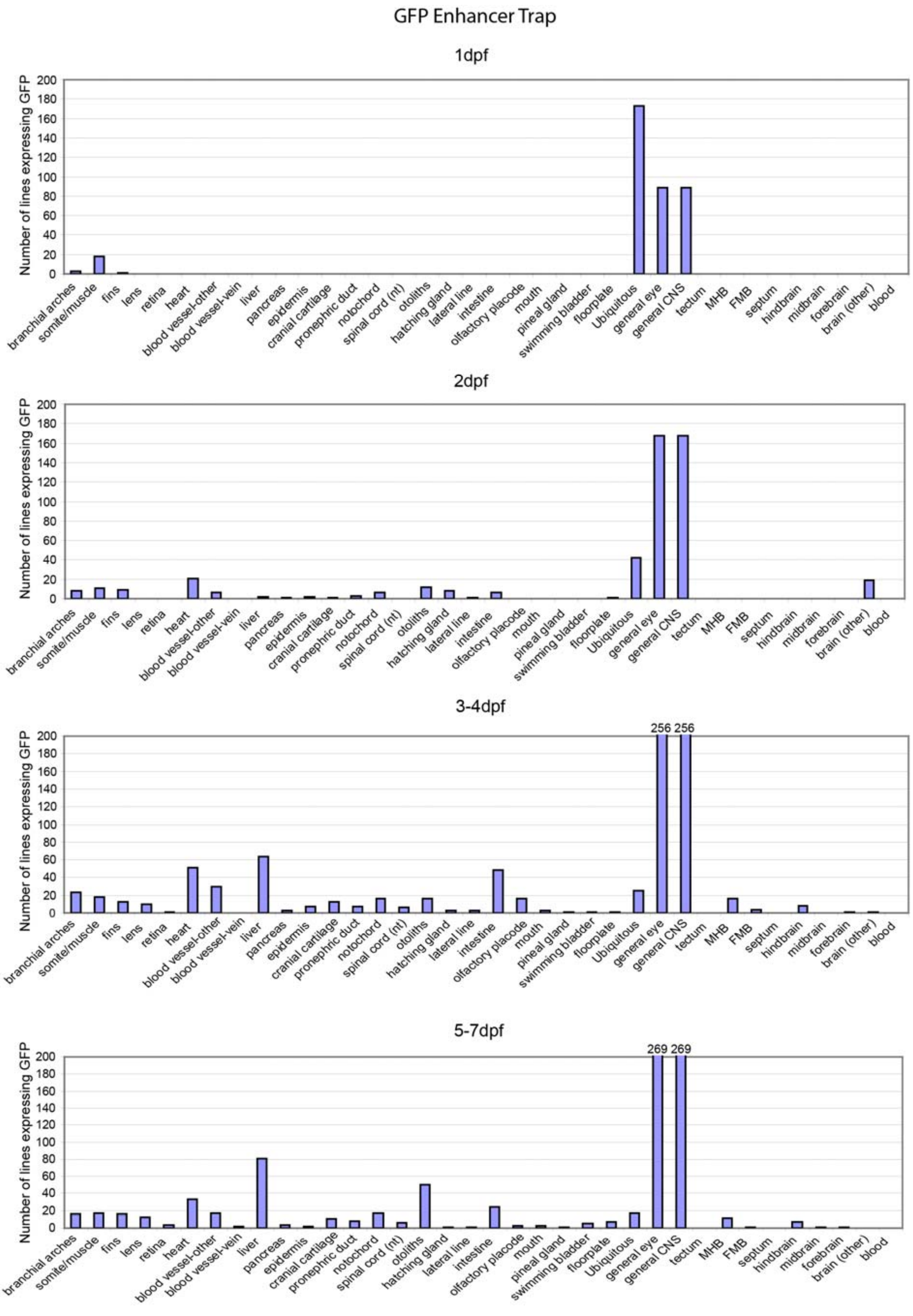

Figure S2 The spatial-temporal distribution of enhancer trap reporter expression. Graphs show the number of lines expressing GFP in various tissues at 1 dpf, 2 dpf, 3-4 dpf and 5-7 dpf. CNS: central nervous system, MHB: mid-hindbrain boundary, FMB: forebrain-midbrain boundary.

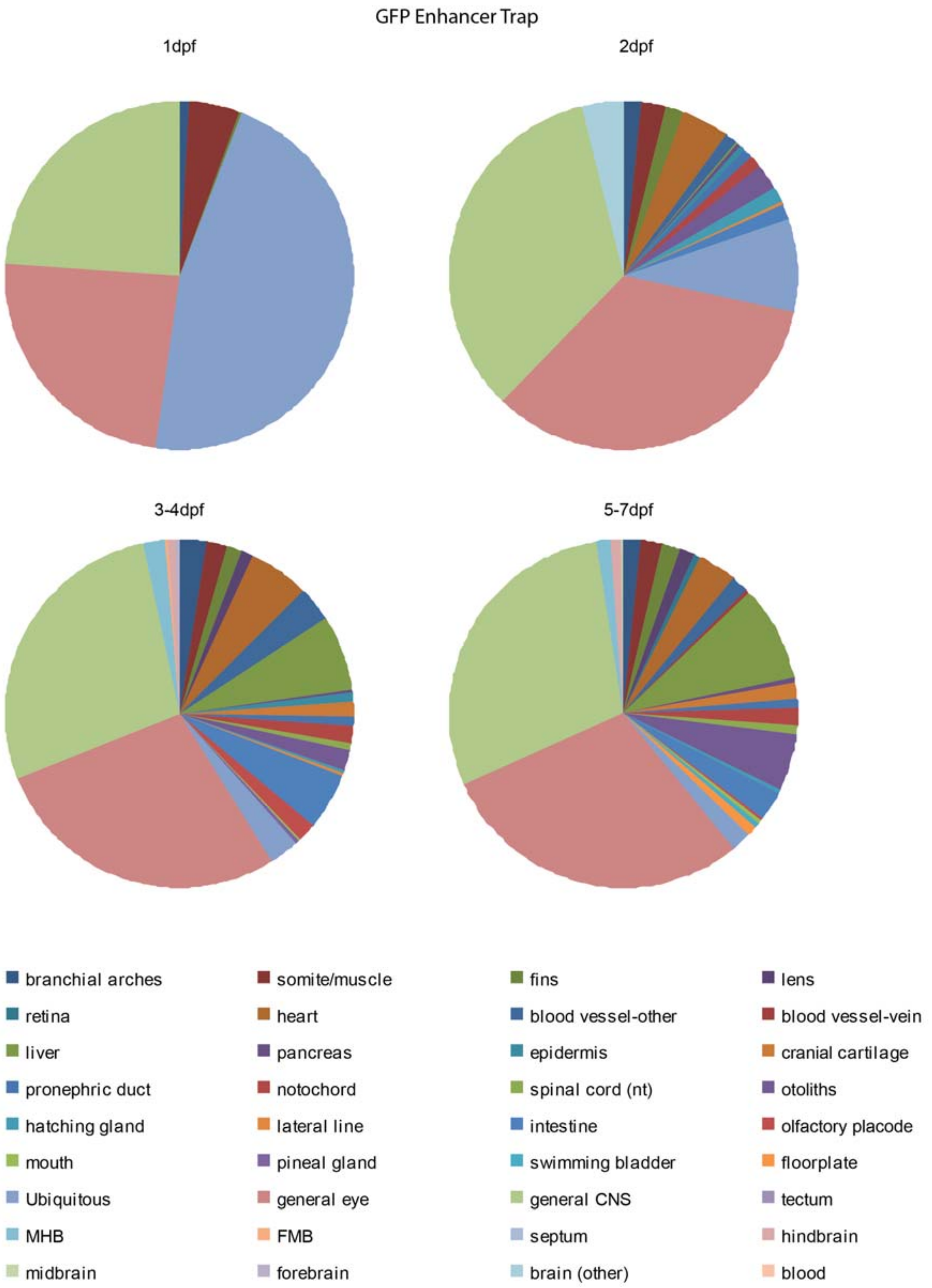

Figure S3 Dynamic expression of the enhancer trap reporter in various tissues. The pie charts show the relative number of lines expressing GFP in various tissues at 1 dpf, 2 dpf, 3-4 dpf and 5-7 dpf. CNS: central nervous system, MHB: mid-hindbrain boundary, FMB: forebrain-midbrain boundary.

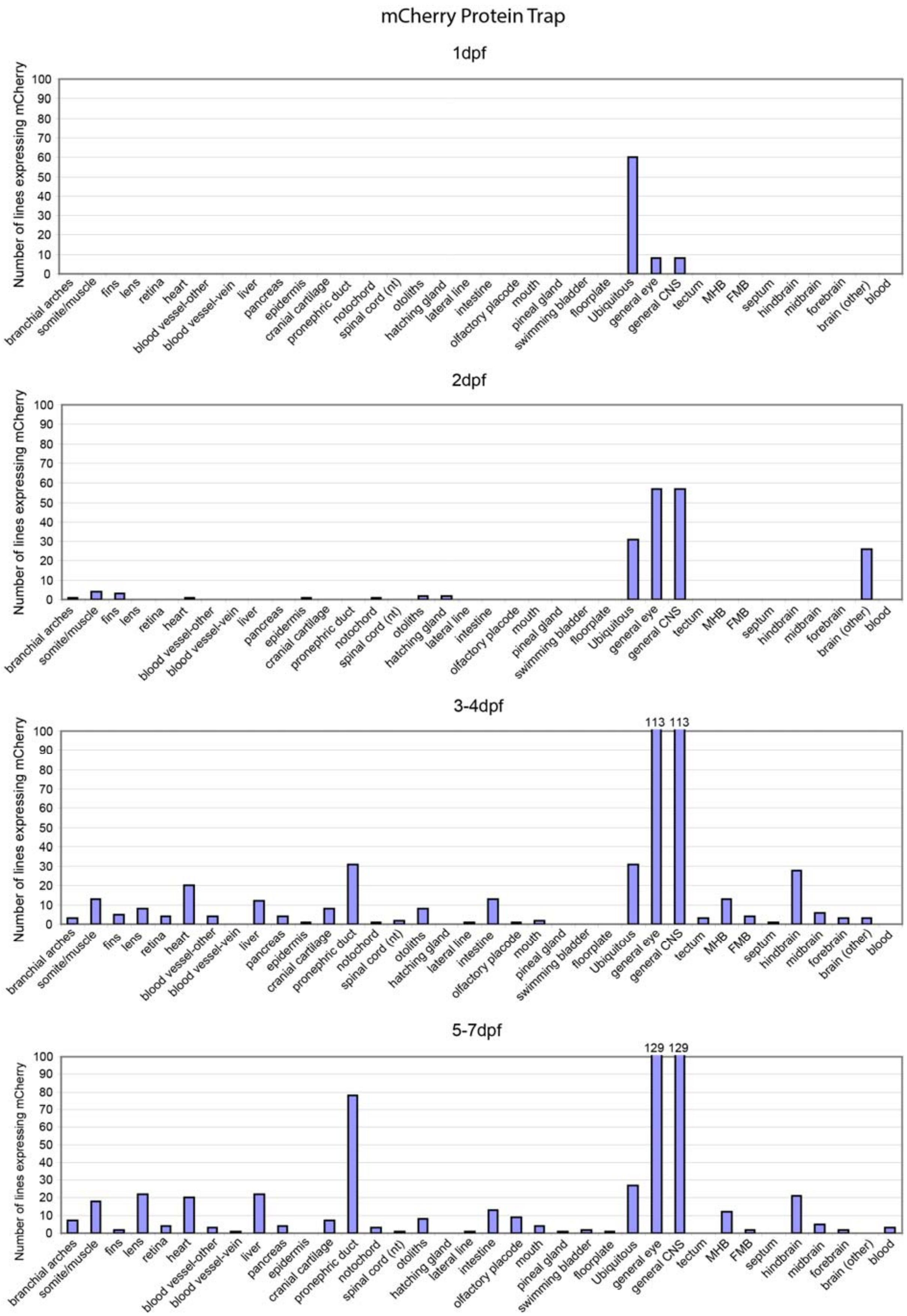

Figure S4 The spatial-temporal distribution of protein trap reporter expression. Graphs show the number of lines expressing mCherry in various tissues at 1 dpf, 2 dpf, 3-4 dpf and 5-7 dpf. CNS: central nervous system, MHB: mid-hindbrain boundary, FMB: forebrain-midbrain boundary.

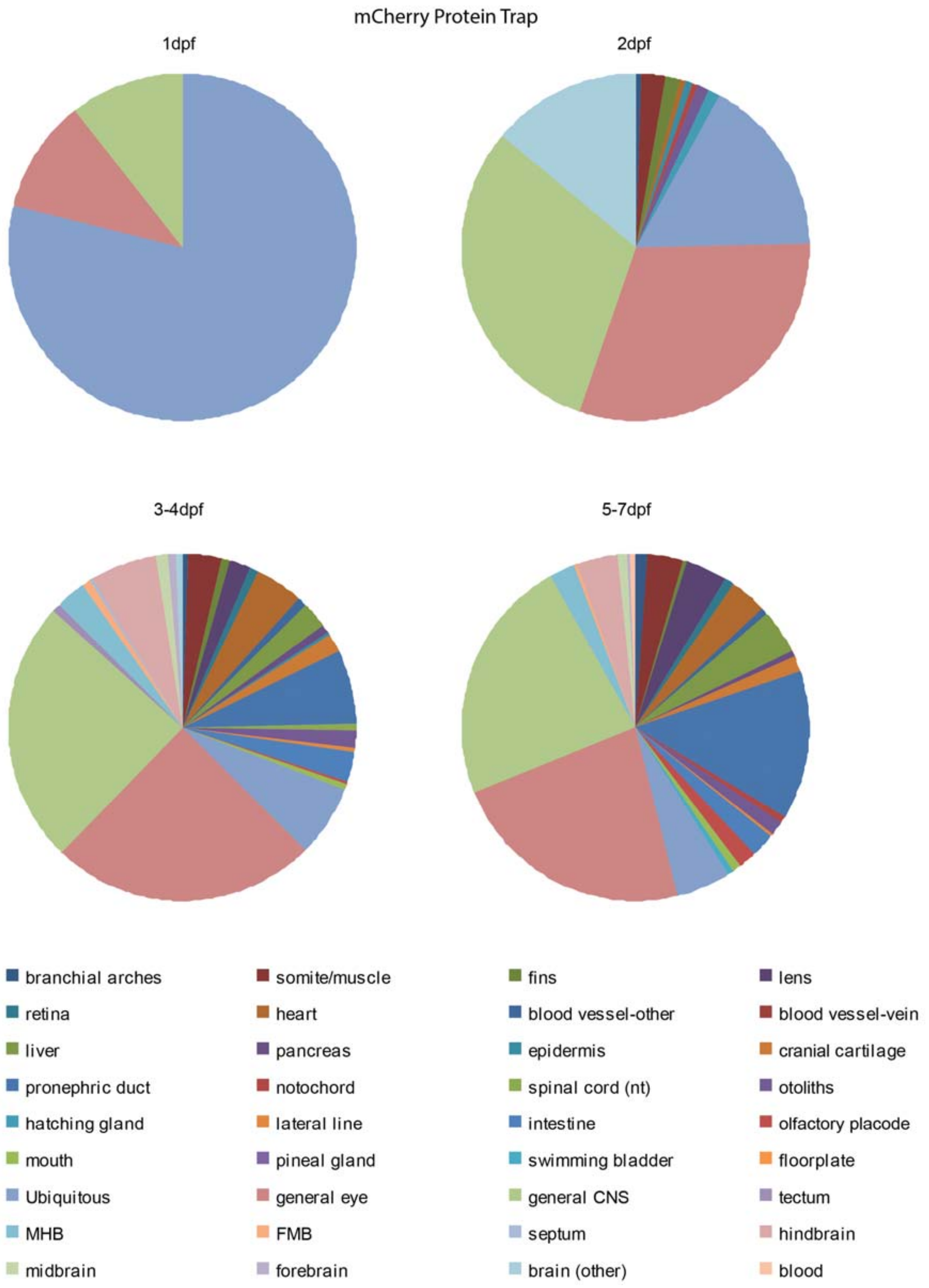

Figure S5 Dynamic expression of the protein trap reporter in various tissues. The pie charts show the relative number of lines expressing mCherry in various tissues at 1 dpf, 2 dpf, 3-4 dpf and 5-7 dpf. CNS: central nervous system, MHB: mid-hindbrain boundary, FMB: forebrain-midbrain boundary.

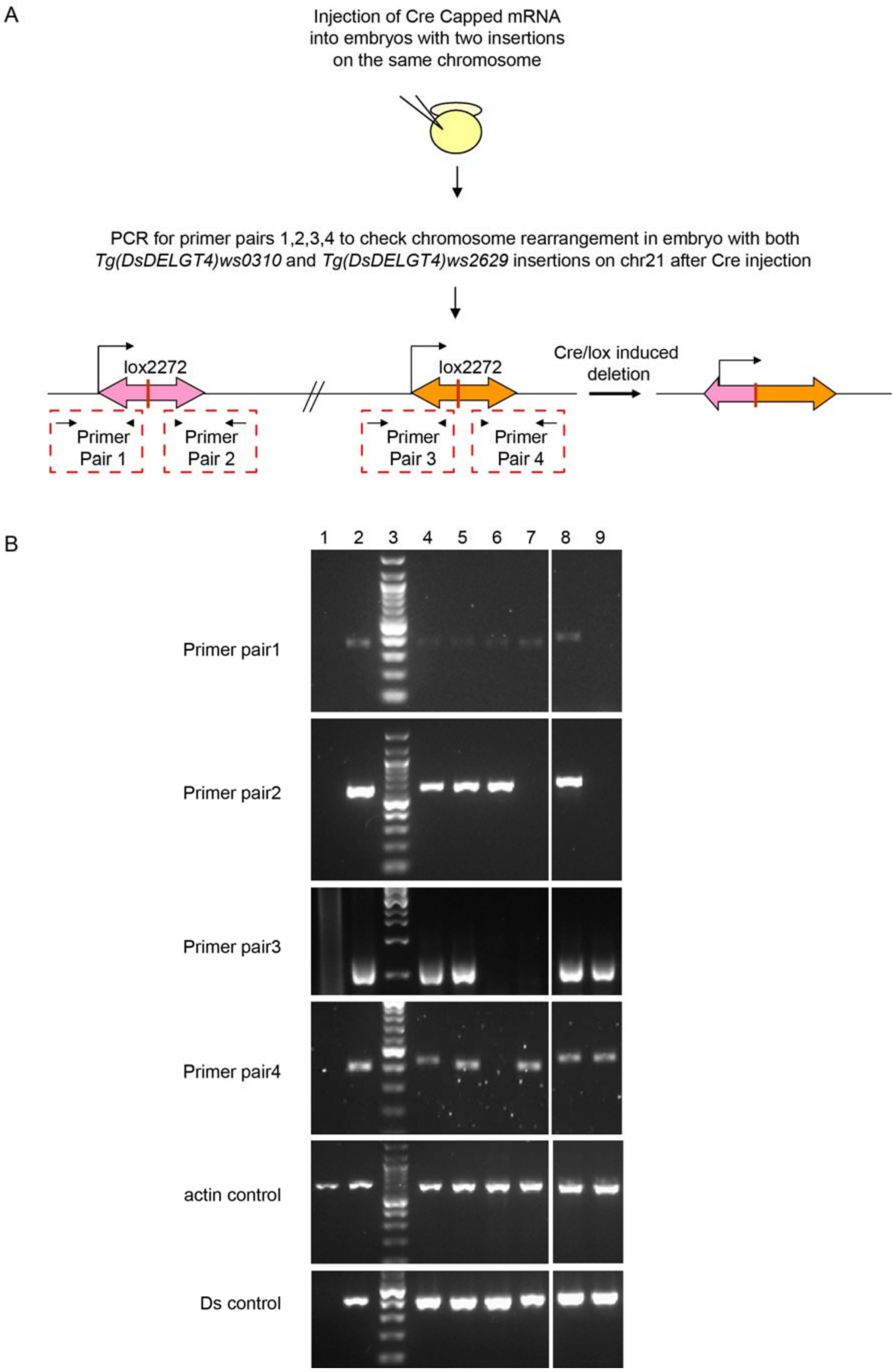
A
Injection of Cre Capped mRNA
into embryos with two insertions
on the same chromosome
PCR for primer pairs 1,2,3,4 to check chromosome rearrangement in embryo with both
Tg(DsDELGT4)ws0310 and Tg(DsDELGT4)ws2629 insertions on chr21 after Cre injection
lox2272
lox2272
Cre/lox induced
deletion
Primer
Pair 1
Primer
Pair 2
Primer
Pair 3
Primer
Pair 4
B
1 2 3 4 5 6 7 8 9
Primer pair1
Primer pair2
Primer pair3
Primer pair4
actin control
Ds control

Figure S6 Cre-lox mediated targeted large deletion. (A) Schematic of a Cre-induced recombination experiment. Cre recombinase capped mRNA was injected into a batch of one-cell stage embryos harboring two insertions on the same chromosome. Individual injected embryos were collected for PCR with six pair of primers to detect deletion events in embryos. In this experiment, the injected embryos carry two insertions *Tg(DsDELGT4)ws0310* and *Tg(DsDELGT4)ws2629* on chr21, 25Mb away from each other. (B) PCR results with six pairs of primers shows the expected deletion in embryo 4 (lane 7). Lane 1 shows PCR results of a wild-type embryo for each primer pair, Lane 2 shows PCR positive control for each primer pair. Lane 3 shows ladders. Lanes 4-9 show PCR products with each primer pair for injected embryos 1-6. Images showing PCR products from embryo 5 and embryo 6 (lanes 8,9) were cropped from a different part of the same gel.

Tables S1-S20

Available for download as Excel files at www.g3journal.org/lookup/suppl/doi:10.1534/g3.114.015842/-/DC1

Table S1 DsDELGT4 lines with reporter expression in liver from day0 to day7

Table S2 DsDELGT4 lines with reporter expression in pancreas from day0 to day7

Table S3 DsDELGT4 lines with reporter expression in intestine from day0 to day7

Table S4 DsDELGT4 lines with reporter expression in swim bladder from day0 to day7

Table S5 DsDELGT4 lines with reporter expression notochord from day0 to day7

Table S6 DsDELGT4 lines with reporter expression in pronephric duct from day0 to day7

Table S7 DsDELGT4 lines with reporter expression in skeletal muscle cells from day0 to day7

Table S8 DsDELGT4 lines with reporter expression in heart tube from day0 to day7

Table S9 DsDELGT4 lines with reporter expression in blood vessels from day0 to day7

Table S10 DsDELGT4 lines with reporter expression in blood cells from day0 to day7

Table S11 DsDELGT4 lines with reporter expression in fin from day0 to day7

Table S12 DsDELGT4 Lines with reporter expression in epidermis from day0 to day7

Table S13 DsDELGT4 lines with reporter expression in mouth from day0 to day7

Table S14 DsDELGT4 lines with reporter expression in olfactory placode from day0 to day7

Table S15 DsDELGT4 lines with reporter expression in cranial cartilage from day0 to day7

Table S16 DsDELGT4 lines with reporter expression in pineal gland from day0 to day7

Table S17 DsDELGT4 lines with reporter expression in lateral line from day0 to day7

Table S18 DsDELGT4 lines with reporter expression in otoliths (ear) from day0 to day7

Table S19 DsDELGT4 lines with reporter expression in branchial arches from day0 to day7

Table S20 Gene ontology analysis.
