# Supplementary material for: A Multifunctional Mutagenesis System for Analysis of Gene Function in Zebrafish
Source: G3 (Bethesda). 2015 Apr 2;5(6):1283–99. doi: 10.1534/g3.114.015842 (PMC4478556; doi:10.1534/g3.114.015842)
Supplement: Supporting Information [file supp_g3.114.015842_FigureS1.pdf]

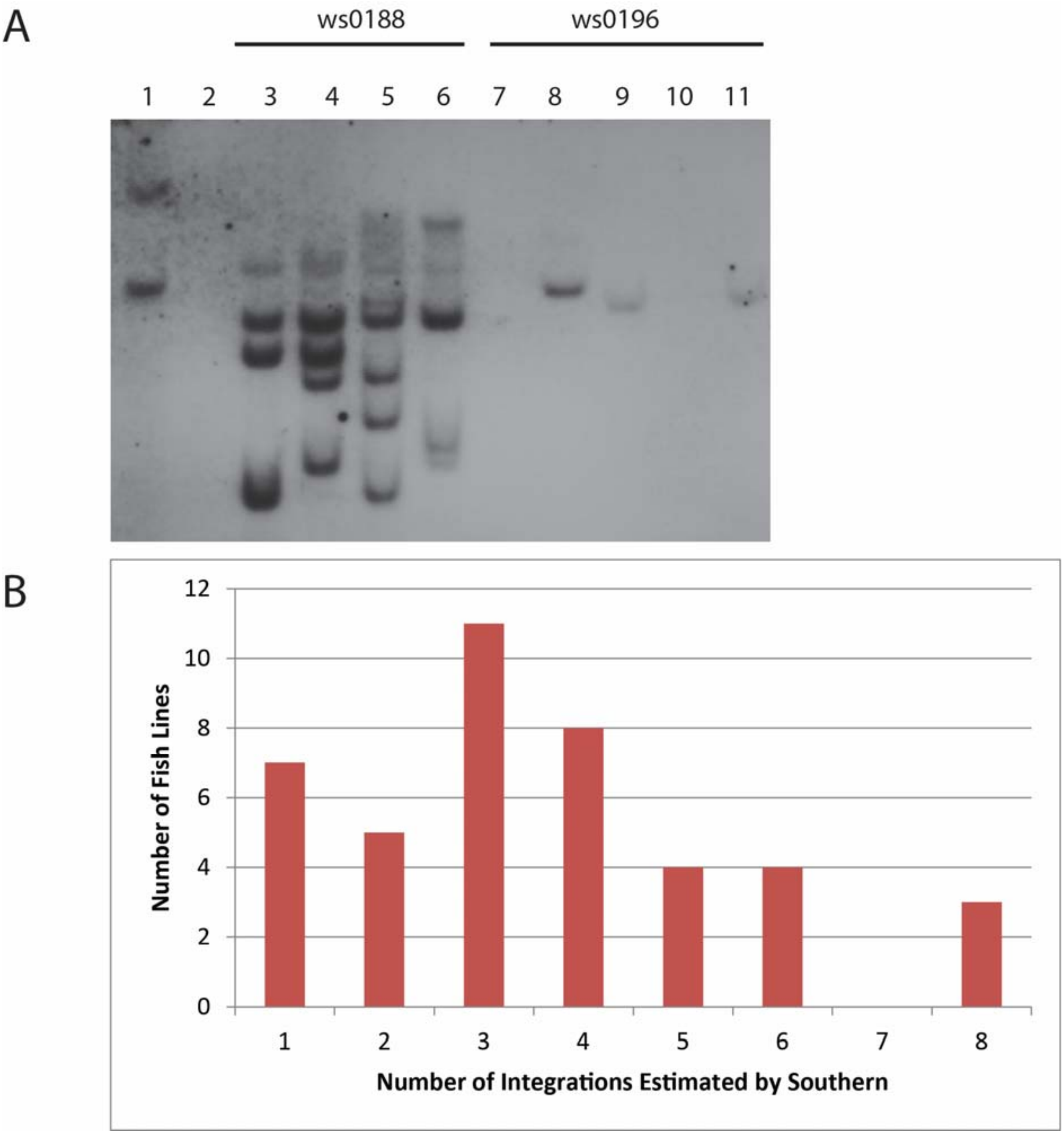


**Figure S1** Number of inserts per line. Southern analysis was performed on 42 representative trapped lines to estimate the number of inserts per line. A) Representative Southern blot analysis. Lane 1: Positive control containing vector DNA and AB genomic DNA. Lane 2: Negative control containing AB genomic DNA. Lanes 3-11: Outcrosses lines *Tg(DsDELGT4)ws0188* and *Tg(DsDELGT4)ws0196* showing multiple integrations per line. B) Estimated number of inserts per line.
