# Supplementary material for: A Multifunctional Mutagenesis System for Analysis of Gene Function in Zebrafish
Source: G3 (Bethesda). 2015 Apr 2;5(6):1283–99. doi: 10.1534/g3.114.015842 (PMC4478556; doi:10.1534/g3.114.015842)
Supplement: Supporting Information [file supp_g3.114.015842_FigureS4.pdf]

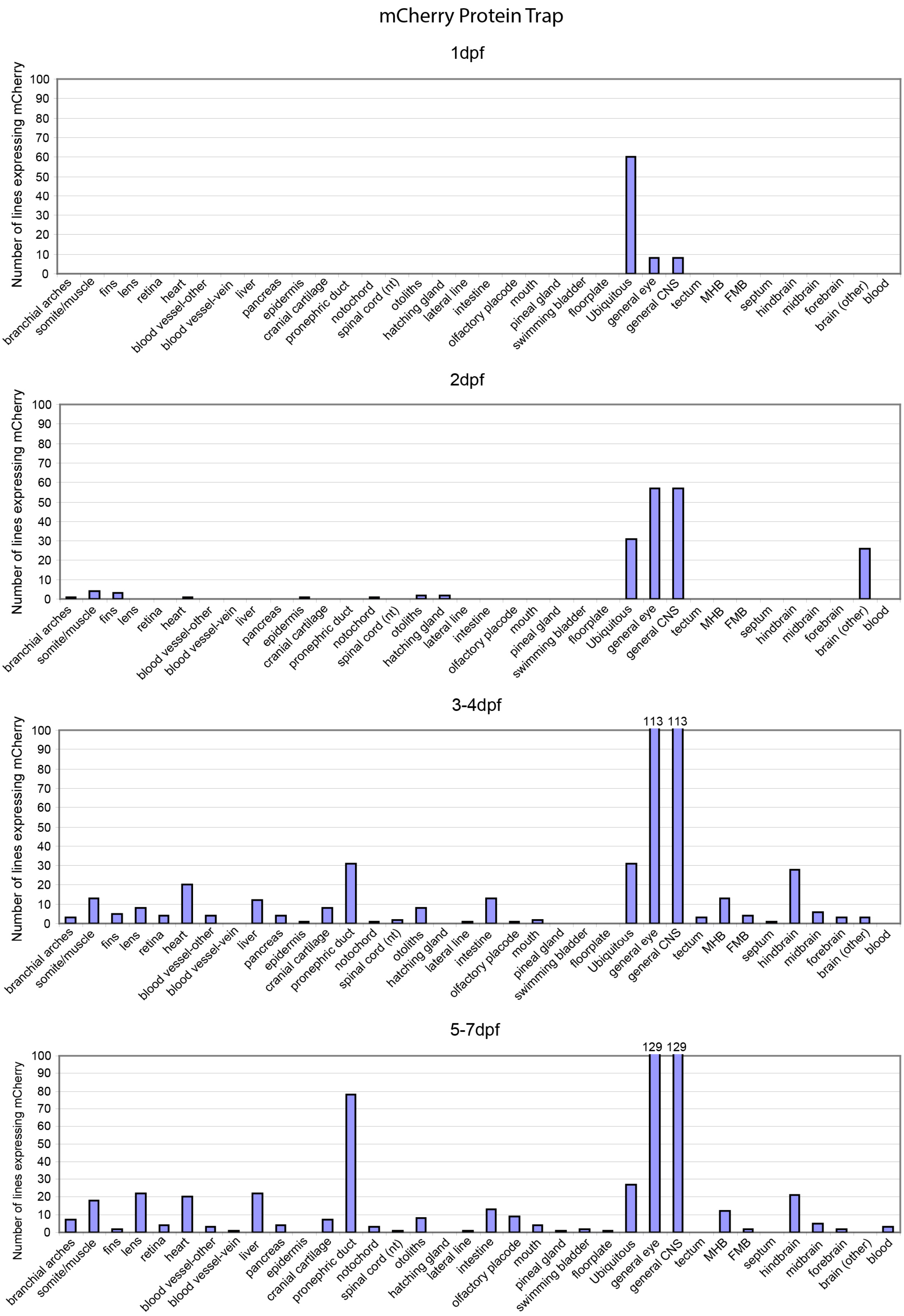

Figure S4 The spatial-temporal distribution of protein trap reporter expression. Graphs show the number of lines expressing mCherry in various tissues at 1 dpf, 2 dpf, 3-4 dpf and 5-7 dpf. CNS: central nervous system, MHB: mid-hindbrain boundary, FMB: forebrain-midbrain boundary.
