# Supplementary material for: A Multifunctional Mutagenesis System for Analysis of Gene Function in Zebrafish
Source: G3 (Bethesda). 2015 Apr 2;5(6):1283–99. doi: 10.1534/g3.114.015842 (PMC4478556; doi:10.1534/g3.114.015842)
Supplement: Supporting Information [file supp_g3.114.015842_FigureS6.pdf]

A

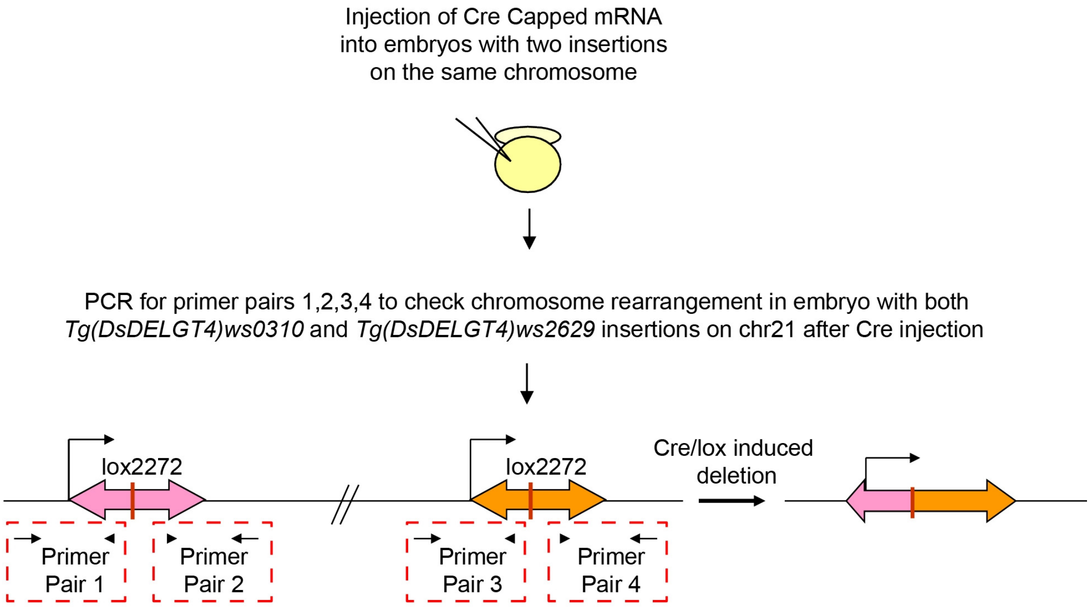
Injection of Cre Capped mRNA
into embryos with two insertions
on the same chromosome
PCR for primer pairs 1,2,3,4 to check chromosome rearrangement in embryo with both
Tg(DsDELGT4)ws0310 and *Tg(DsDELGT4)ws2629* insertions on chr21 after Cre injection
lox2272
lox2272
Cre/lox induced
deletion
Primer
Pair 1
Primer
Pair 2
Primer
Pair 3
Primer
Pair 4

B

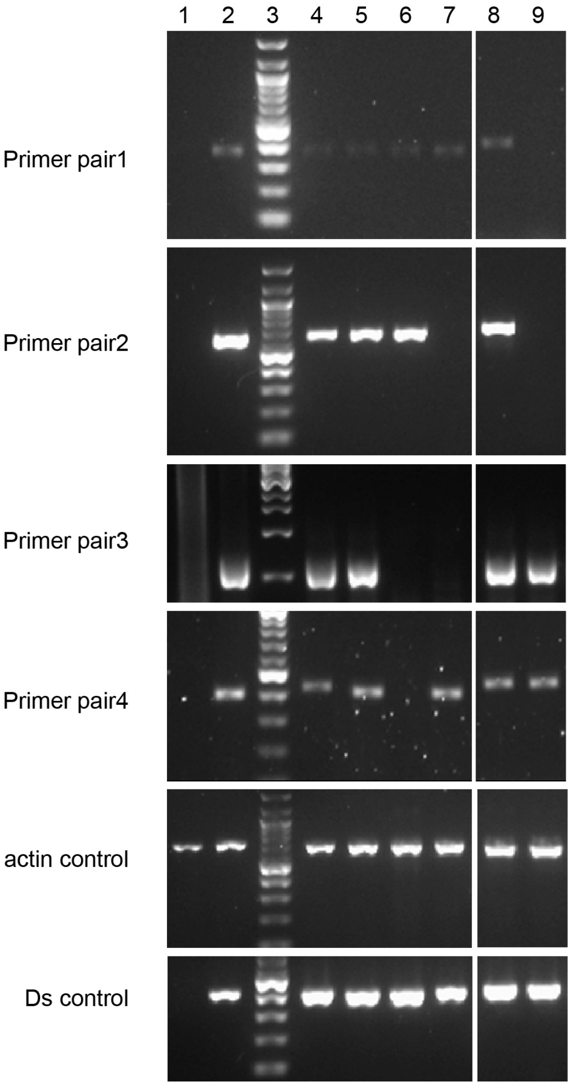
1
2
3
4
5
6
7
8
9
Primer pair1
Primer pair2
Primer pair3
Primer pair4
actin control
Ds control

Figure S6 Cre-lox mediated targeted large deletion. (A) Schematic of a Cre-induced recombination experiment. Cre recombinase capped mRNA was injected into a batch of one-cell stage embryos harboring two insertions on the same chromosome. Individual injected embryos were collected for PCR with six pair of primers to detect deletion events in embryos. In this experiment, the injected embryos carry two insertions *Tg(DsDELGT4)ws0310* and *Tg(DsDELGT4)ws2629* on chr21, 25Mb away from each other. (B) PCR results with six pairs of primers shows the expected deletion in embryo 4 (lane 7). Lane 1 shows PCR results of a wild-type embryo for each primer pair, Lane 2 shows PCR positive control for each primer pair. Lane 3 shows ladders. Lanes 4-9 show PCR products with each primer pair for injected embryos 1-6. Images showing PCR products from embryo 5 and embryo 6 (lanes 8,9) were cropped from a different part of the same gel.
